# Characteristics Associated With Racial/Ethnic Disparities in COVID-19 Outcomes in an Academic Health Care System

**DOI:** 10.1001/jamanetworkopen.2020.25197

**Published:** 2020-10-21

**Authors:** Tian Gu, Jasmine A. Mack, Maxwell Salvatore, Swaraaj Prabhu Sankar, Thomas S. Valley, Karandeep Singh, Brahmajee K. Nallamothu, Sachin Kheterpal, Lynda Lisabeth, Lars G. Fritsche, Bhramar Mukherjee

**Affiliations:** 1 Department of Biostatistics, University of Michigan School of Public Health, Ann Arbor; 2 Rogel Cancer Center, University of Michigan Medicine, Ann Arbor; 3 Data Office for Clinical and Translational Research, University of Michigan, Ann Arbor; 4 Division of Pulmonary and Critical Care Medicine, Department of Internal Medicine, University of Michigan Medical School, Ann Arbor; 5 Institute for Healthcare Policy and Innovation, University of Michigan, Ann Arbor; 6 Department of Learning Health Sciences, University of Michigan, Ann Arbor; 7 Division of Cardiovascular Medicine, Department of Internal Medicine, University of Michigan Medical School, Ann Arbor; 8 Department of Anesthesiology, University of Michigan Medical School, Ann Arbor; 9 Department of Epidemiology, University of Michigan School of Public Health, Ann Arbor; 10 Center for Statistical Genetics, University of Michigan School of Public Health, Ann Arbor

## Abstract

**Importance:**

Black patients are overrepresented in the number of COVID-19 infections, hospitalizations, and deaths in the US. Reasons for this disparity may be due to underlying comorbidities or sociodemographic factors that require further exploration.

**Objective:**

To systematically determine patient characteristics associated with racial/ethnic disparities in COVID-19 outcomes.

**Design, Setting, and Participants:**

This retrospective cohort study used comparative groups of patients tested or treated for COVID-19 at the University of Michigan from March 10, 2020, to April 22, 2020, with an outcome update through July 28, 2020. A group of randomly selected untested individuals were included for comparison. Examined factors included race/ethnicity, age, smoking, alcohol consumption, comorbidities, body mass index (BMI; calculated as weight in kilograms divided by height in meters squared), and residential-level socioeconomic characteristics.

**Exposure:**

In-house polymerase chain reaction (PCR) tests, commercial antibody tests, nasopharynx or oropharynx PCR deployed by the Michigan Department of Health and Human Services and reverse transcription–PCR tests performed in external labs.

**Main Outcomes and Measures:**

The main outcomes were being tested for COVID-19, having test results positive for COVID-19 or being diagnosed with COVID-19, being hospitalized for COVID-19, requiring intensive care unit (ICU) admission for COVID-19, and COVID-19–related mortality (including inpatient and outpatient). Medical comorbidities were defined from the *International Classification of Diseases, Ninth Revision*, and *International Classification of Diseases, Tenth Revision*, codes and were aggregated into a comorbidity score. Associations with COVID-19 outcomes were examined using odds ratios (ORs).

**Results:**

Of 5698 patients tested for COVID-19 (mean [SD] age, 47.4 [20.9] years; 2167 [38.0%] men; mean [SD] BMI, 30.0 [8.0]), most were non-Hispanic White (3740 patients [65.6%]) or non-Hispanic Black (1058 patients [18.6%]). The comparison group included 7168 individuals who were not tested (mean [SD] age, 43.1 [24.1] years; 3257 [45.4%] men; mean [SD] BMI, 28.5 [7.1]). Among 1139 patients diagnosed with COVID-19, 492 (43.2%) were White and 442 (38.8%) were Black; 523 (45.9%) were hospitalized, 283 (24.7%) were admitted to the ICU, and 88 (7.7%) died. Adjusting for age, sex, socioeconomic status, and comorbidity score, Black patients were more likely to be hospitalized compared with White patients (OR, 1.72 [95% CI, 1.15-2.58]; *P* = .009). In addition to older age, male sex, and obesity, living in densely populated areas was associated with increased risk of hospitalization (OR, 1.10 [95% CI, 1.01-1.19]; *P* = .02). In the overall population, higher risk of hospitalization was also observed in patients with preexisting type 2 diabetes (OR, 1.82 [95% CI, 1.25-2.64]; *P* = .02) and kidney disease (OR, 2.87 [95% CI, 1.87-4.42]; *P* < .001). Compared with White patients, obesity was associated with higher risk of having test results positive for COVID-19 among Black patients (White: OR, 1.37 [95% CI, 1.01-1.84]; *P* = .04. Black: OR, 3.11 [95% CI, 1.64-5.90]; *P* < .001; *P *for interaction = .02). Having any cancer was associated with higher risk of positive COVID-19 test results for Black patients (OR, 1.82 [95% CI, 1.19-2.78]; *P* = .005) but not White patients (OR, 1.08 [95% CI, 0.84-1.40]; *P* = .53; *P *for interaction = .04). Overall comorbidity burden was associated with higher risk of hospitalization in White patients (OR, 1.30 [95% CI, 1.11-1.53]; *P* = .001) but not in Black patients (OR, 0.99 [95% CI, 0.83-1.17]; *P* = .88; *P *for interaction = .02), as was type 2 diabetes (White: OR, 2.59 [95% CI, 1.49-4.48]; *P* < .001; Black: OR, 1.17 [95% CI, 0.66-2.06]; *P* = .59; *P *for interaction = .046). No statistically significant racial differences were found in ICU admission and mortality based on adjusted analysis.

**Conclusions and Relevance:**

These findings suggest that preexisting type 2 diabetes or kidney diseases and living in high–population density areas were associated with higher risk for COVID-19 hospitalization. Associations of risk factors with COVID-19 outcomes differed by race.

## Key Points

**Question:**

What sociodemographic and underlying health conditions are associated with COVID-19 outcomes and do they differ by race/ethnicity?

**Findings:**

In this cohort study of 5698 patients tested for or diagnosed with COVID-19, high population density, type 2 diabetes, and kidney disease were associated with hospitalization, in addition to older age, male sex, and obesity. Adjusting for covariates, non-Hispanic Black patients were 1.72-fold more likely to be hospitalized than non-Hispanic White patients, while no significant race differences were observed in intensive care unit admission and mortality.

**Meaning:**

These findings suggest that racial disparities existed in COVID-19 outcomes that cannot be explained after controlling for age, sex, socioeconomic status, and comorbidity score; therefore, targeted interventions to support high-risk populations are needed.

## Teaser

This cohort study examines sociodemographic characteristics associated with coronavirus disease 2019 outcomes among Black and White patients in Michigan.

## Introduction

The coronavirus disease 2019 (COVID-19) pandemic, caused by severe acute respiratory syndrome coronavirus 2 (SARS-CoV-2), has underscored racial disparities in the United States.^1,2,3,4,5,6,7,8,9,10,11,12,13,14,15,16,17,18,19,20,21,22,23,24,25^ In the state of Michigan in particular, there have been 113 820 confirmed COVID-19 cases and 6767 deaths as of September 1, 2020, which makes Michigan one of the most affected states in the US.^26^ While Black residents represent 14% of the Michigan population,^27^ they account for 21% of COVID-19 cases and 37% of deaths attributed to COVID-19.^26^ Similar trends have been observed in New York^9^ and other states, where there is an overrepresentation of Black and Latinx people in COVID-19 cases and deaths.^23^

Overrepresentation of minority populations in worse COVID-19 outcomes may be explained by a myriad of factors, such as by weathering (ie, early health deterioration due to cumulative impact of socioeconomic disparity),^28,29,30^ higher comorbidity burden,^31^ inadequate health care,^31^ and socioeconomic differences related to unemployment, food insecurity, and housing instability.^29,30^ Several studies have reported that individuals who are members of racial/ethnic minority groups, men, older, and current smokers, and those who have comorbidity burdens have higher risk of COVID-19 susceptibility and hospitalization.^2,14,16,32,33,34,35,36^ Individuals in racial/ethnic minority groups who maintain livelihoods as essential workers are more likely to be exposed to SARS-CoV-2,^23,25^ whereas living in high density areas,^1^ homelessness,^37^ and incarceration^38^ add to the barriers to social distancing.^15,23^

Although studies have reported some explanation for these disparities, the substantial evidence supporting the observed disparity in COVID-19 outcomes with appropriate covariates and comparison groups remains limited,^17^ which calls for in-depth studies to explain the underlying reasons while controlling for confounders, such as socioeconomic status.^7,22,24,29,39,40^ In addition, experiences from COVID-19 highlight the need to not only identify risk factors, but also to avoid spurious conclusions of racial/ethnic differences being explained by biology, which could further perpetuate racial/ethnic stereotypes.^29^ Additionally, some previous studies have compared individuals with test results positive for COVID-19 with those with test results negative for COVID-19, instead of population-based comparative groups, in which selection bias is potentially at play.^41,42^

The objective of this study is to systematically determine sociodemographic characteristics and comorbid conditions that are associated with COVID-19 outcomes (ie, having positive test results, hospitalization, admission to ICU, and mortality) by race/ethnicity, using electronic health records (EHRs) from the University of Michigan Health System, also known as Michigan Medicine (MM), which serves a large patient population in the US Midwest.

## Methods

The University of Michigan Medical School institutional review board reviewed the study and determined that it is exempt. The institutional review board waived the need for ethics approval and the need to obtain consent for the collection, analysis, and publication of the anonymized COVID-19 data, per institutional policy. This study followed the Strengthening the Reporting of Observational Studies in Epidemiology (STROBE) reporting guideline.

### COVID-19 Cohort

We extracted the EHR data for patients with test results for COVID-19 at MM. Our study cohort consisted of 5698 tested or diagnosed patients, including 5548 patients who were tested at MM from March 10, 2020, to April 22, 2020, 119 transfer patients from other hospitals, and 31 patients who were tested elsewhere but treated at MM. The selected cohort is a nonrandom sample of the population, since the testing protocol at MM focused on prioritized testing^43^ (eg, testing symptomatic individuals and those at the highest risk of exposure). Our COVID-19–positive cohort contained 1139 patients whose test results were positive for SARS-CoV-2. We updated the COVID-19 outcomes of the study cohort through July 28, 2020.

### COVID-19 Testing

Four types of diagnostic tests were used in the tested cohort at MM, including an in-house polymerase chain reaction (PCR) test (5051 patients [88.6%]), a commercial antibody test (Viracor; 419 patients [7.4%]), COVID-19 nasopharynx or oropharynx PCR tests deployed by the Michigan Department of Health and Human Services (55 patients [1.0%]), and a small fraction of reverse transcription–PCR tests performed in external labs (13 patients [0.2%]); 160 tested patients (2.8%) were transferred, tested elsewhere, or had no information on type of testing they received.

### Comparative Group Selection

To understand how selection bias factored into our tested sample, in addition to comparing patients with COVID-19 with those whose test results were negative, we created an untested comparison group of 7168 individuals from the MM database, a similar-sized random sample of contemporaneous patients. We initially extracted 20 000 random individuals before limiting the group to 7211 patients who were alive at the time of data extraction (ie, April 22, 2020) and have had encounters with the health system after April 22, 2012. At the time of updating COVID-19 outcomes (July 28, 2020), we further excluded 43 patients who had been tested since the initial data extraction and achieved the final sample size of 7168 individuals in the comparison group.

### COVID-19 Outcomes and Description of Variables

The eFigure in the Supplement presents a flow diagram of sample sizes corresponding to each COVID-19 outcome used in this study. A summary data dictionary is available with source and definition of each variable used in our analysis in eTable 1 in the Supplement. In addition to being tested for and having test results positive for COVID-19, among the COVID-19–positive cohort, we considered various stages of progression of the disease based on admission and discharge data, including hospitalization, intensive care unit (ICU) care, and death. Hospitalizations were defined as inpatients with COVID-19 diagnosis, for whom the admission date was within the time frame of the data update (March 10 to July 28, 2020). Patients who received ICU care were defined as patients who were admitted to the ICU any time during their COVID-19–related hospitalization. Mortality data, including inpatient and nonhospitalized deaths, were extracted from patient EHRs, defined as death that had occurred after a confirmed positive result for a COVID-19 laboratory test.

### Classifying Patients Who Were Still in the Hospital and ICU

A total of 21 patients were still admitted in the hospital (3 patients) or in an ICU (19 patients) at the time of the data update (July 28, 2020). A sensitivity analysis showed similar results after excluding these patients whose final outcome was unclear (ie, still in the hospital or ICU) from the analysis.

### Generation of Comorbidities From EHRs

Based on Centers for Disease Control and Prevention guidelines on risk factors for COVID-19^44^ and previous studies,^34,45,46^ we constructed COVID-19–related comorbid conditions using available *International Classification of Diseases, Ninth Revision* and *Tenth Revision* codes for 12 036 individuals (tested or diagnosed: 5225 individuals; untested comparison group: 6811 individuals) from their EHRs. Longitudinal time-stamped diagnoses were recoded as indicator variables for whether a patient ever had a given diagnosis code recorded by MM. To differentiate preexisting conditions from diagnoses related to COVID-19 testing or treatment, we applied a 14-day-prior restriction on the tested cohort by removing diagnoses that first appeared within the 14 days before the first test or diagnosis date, whichever was earlier (4998 of the 5225 tested individuals had diagnoses data after enforcing the 14-days-prior restriction). We focused on 7 binary disease indicators that have been specifically reported in relation to COVID-19 outcomes^34,35,36,44,47,48^: respiratory conditions, circulatory conditions, any cancer, type 2 diabetes, kidney disease, liver disease, and autoimmune disease (eTable 1 in the Supplement). We calculated a comorbidity score as the sum of these 7 items that ranges from 0 to 7. This score was used as an adjustment or risk factor capturing the general health status.

### Defining Socioeconomic Status and Other Adjustment Covariates

Self-reported sex, race/ethnicity, smoking status, alcohol consumption, body mass index (BMI; calculated as weight in kilograms divided by height in meters squared), and age were extracted from the EHRs. We classified patients to be seeking primary care in MM if they had at least 1 encounter in any of the primary care locations in MM since January 1, 2018. Measures of socioeconomic characteristics are defined by US census tract (based on residential address available in each patient’s EHR) for the year 2010. The boundaries for the census tracts were normalized to 2010 tract boundaries using the Longitudinal Tract Data Base.^49^ Following Clarke et al,^50^ we evaluated 3 composite indices: neighborhood disadvantage, neighborhood affluence, and ethnic and immigrant concentration, calculated routinely in the National Neighborhood Data Archive, a publicly available data source, to measure physical and social environment.^51^ Because the results that adjusted for all 3 indices were similar to the results that adjusted for only the first index, the 2010 Neighborhood Socioeconomic Disadvantage Index (NDI), without proportion of Black residents, we used the NDI as a marker of neighborhood socioeconomic status in this study. The NDI is defined as the mean of the proportion of the population that is in poverty, unemployed, using public assistance income, and woman-headed families with children. We also included population density (in persons per square mile) as a covariate in the susceptibility models.^51^

### Statistical Analysis

Since all outcomes were binary, we performed logistic regression to assess the risk factors of COVID-19 outcomes. We reported Firth bias-corrected estimates of the odds ratio (OR) to address potential separation issues, with their corresponding 95% Wald-type CI and *P* value. Four nested covariate adjustments were explored to check the robustness of inference to the choice of potential confounders (eTable 1 and eTable 2 in the Supplement). The final adjustment model we used included age, sex, race/ethnicity, population density, NDI, and comorbidity score. We used population density only in the getting tested and susceptibility models, and we refrained from using the composite comorbidity score when examining associations with individual comorbidities.

The analysis model is:

*logit P*(*Y_COVID_* = *1|X*, *Covariate*) = *β_0_* + *β_X_X* + *β_cov_Covariate*

in which *X* is the variable or risk factor of interest, and *Covariate* denotes the vector of covariates. Here, *Y_COVID_* is 3 different types of COVID-19–related outcomes under consideration: (1) characteristics associated with being tested, comparing the tested cohort with those who were not tested for COVID-19 (using a randomly selected untested comparison group); (2) risk factors of COVID-19 susceptibility, comparing the COVID-19–positive cohort with those who were not diagnosed with COVID-19 (using an untested comparison group and the tested negative comparison group), and (3) risk factors of COVID-19 outcomes (no comparison group involved), examined among the COVID-19–positive cohort comparing those who were hospitalized with those who were not, those who were admitted to an ICU with those who were not, and those who died with those who did not.

In addition, we carried out a set of interaction analyses by race using the following model to evaluate the difference between White and Black patients:

*logit P*(*Y_COVID_* = *1|X*, *Race*, *Covariate*) = *β_0_* + *β_X_X* + *β_Race_Race* + *β_int_X* × *Race* + *β_cov_Covariate*

in which *Race* included 4 categories: White, Black, other known race/ethnicity, and unknown race/ethnicity. We reported the subgroup effects for White and Black patients using this model, as well as their difference by testing *H_0_:β_int_* = 0.

All analyses were performed in R statistical software version 3.6.2 (R Project for Statistical Computing). Statistical significance was defined using a 2-sided significance level of α = .05. Missing data were handled by using a complete case analysis, which leads to unbiased inference when the covariates are missing completely at random. Sensitivity analysis are presented in eTable 3 and eTable 4 in the Supplement.

## Results

### Descriptive Statistics

Our tested cohort included 5698 patients (mean [SD] age, 47.4 [20.9] years; 2167 [38.0%] men; mean [SD] BMI 30 [8.0]), among whom 1139 patients (20.0%) had test results positive for COVID-19 (Table 1). The comparison group included 7168 individuals who were not tested (mean [SD] age, 43.1 [24.1] years; 3257 [45.4%] men; mean [SD] BMI, 28.5 [7.1]). In the tested cohort, 3172 patients (55.7%) received primary care at MM. Most of the tested cohort were either White (3740 patients [65.6%]) or Black (1058 patients [18.6%]). Among 1139 patients with positive COVID-19 test results, 523 (45.9%) were hospitalized, 283 (24.8%) were admitted to an ICU, and 88 (7.7%) died. As the disease progressed among patients with positive COVID-19 test results (from nonhospitalized to hospitalized and ICU), the proportion of patients who were older (ie, ≥65 years), men, with higher BMI, and former or current smoker and who consumed alcohol consistently increased. The descriptive characteristics of both the tested cohort and the COVID-19–positive cohort indicate higher enrichment of underlying medical conditions. Missingness information corresponding to each variable is presented in eTable 5 in the Supplement.

**Table 1.** Descriptive Characteristics of the COVID-19 Tested or Diagnosed Cohort

Variable	Individuals, No. (%)^a^						
	Tested for COVID-19						Comparison group (n = 7168)
	Overall (n = 5698)	Negative results (n = 4559)	Positive results				
			Overall (n = 1139)	Hospitalized (n = 523)	ICU (n = 283)	Deceased (n = 88)	
Age, y							
Mean (SD)	47.4 (20.9)	46.0 (21.3)	53.0 (17.9)	60.6 (16.6)	60.0 (16.5)	71.7 (13.5)	43.1 (24.1)
Median (IQR)	48 (32-63)	46 (32-63)	53 (39-66)	62 (50-73)	62 (51-71)	73 (64-82)	43 (23-63)
<18	373 (6.5)	364 (8.0)	9 (0.8)	4 (0.8)	3 (1.1)	0	1661 (23.2)
18 to <35	1295 (22.7)	1092 (24.0)	203 (17.8)	38 (7.3)	26 (9.2)	2 (2.3)	1254 (17.5)
35 to <50	1364 (23.9)	1107 (24.3)	257 (22.6)	83 (15.9)	36 (12.7)	5 (5.7)	1178 (16.4)
50 to <65	1381 (24.2)	1032 (22.6)	349 (30.6)	171 (32.7)	96 (33.9)	16 (18.2)	1412 (19.7)
65 to <80	926 (16.3)	693 (15.2)	233 (20.5)	154 (29.4)	91 (32.2)	31 (35.2)	1206 (16.8)
≥80	359 (6.3)	271 (5.9)	88 (7.7)	73 (14.0)	31 (11.0)	34 (38.6)	454 (6.3)
Men	2167 (38.0)	1636 (35.9)	531 (46.6)	294 (56.2)	169 (59.7)	56 (63.6)	3257 (45.4)
Primary care in MM	3172 (55.7)	2597 (57.0)	575 (50.5)	161 (30.8)	85 (30.0)	24 (27.3)	1117 (15.6)
BMI							
Mean (SD)	30 (8.0)	29.4 (7.5)	32.2 (9.3)	33.1 (10.8)	33.9 (12.3)	31.8 (7.6)	28.5 (7.05)
<18.5	83 (1.7)	74 (1.9)	9 (0.9)	5 (1.0)	2 (0.7)	1 (1.2)	85 (1.2)
18.5 to <25	1300 (26.3)	1125 (28.9)	175 (16.7)	63 (12.6)	37 (13.5)	13 (15.5)	1329 (18.5)
25 to <30	1476 (29.9)	1157 (29.8)	319 (30.4)	163 (32.7)	81 (29.6)	28 (33.3)	1213 (16.9)
≥30	2077 (42.1)	1531 (39.4)	546 (52.0)	268 (53.7)	154 (56.2)	42 (50.0)	1370 (19.1)
Smoking status							
Never	3144 (61.7)	2506 (60.6)	638 (66.5)	264 (62.0)	114 (55.9)	19 (35.8)	3673 (51.2)
Past	1522 (29.9)	1239 (30.0)	283 (29.5)	153 (35.9)	86 (42.2)	34 (64.2)	1084 (15.1)
Current	427 (8.4)	388 (9.4)	39 (4.1)	9 (2.1)	4 (2.0)	0	572 (8.0)
Ever	1949 (38.3)	1627 (39.4)	322 (33.5)	162 (38.0)	90 (44.1)	34 (64.2)	1656 (23.1)
Alcohol consumption	2774 (66.7)	2302 (67.0)	472 (65.2)	170 (62.5)	90 (65.7)	27 (65.9)	2171 (30.3)
Race/ethnicity							
White	3740 (65.6)	3248 (71.2)	492 (43.2)	190 (36.3)	101 (35.7)	35 (39.8)	3573 (49.8)
Black	1058 (18.6)	616 (13.5)	442 (38.8)	233 (44.6)	132 (46.6)	36 (40.9)	391 (5.5)
Other^b^	544 (9.5)	423 (9.3)	121 (10.6)	51 (9.8)	19 (6.7)	3 (3.4)	531 (7.4)
Unknown^c^	356 (6.2)	272 (6.0)	84 (7.4)	49 (9.4)	31 (11)	14 (15.9)	2673 (37.3)
NDI, mean (SD)	0.11 (0.08)	0.1 (0.08)	0.12 (0.09)	0.13 (0.1)	0.14 (0.1)	0.14 (0.09)	0.11 (0.08)
Population density, persons/square mile	2650 (2337.5)	2541 (2328.6)	3160 (2313.1)	3608 (2564.1)	3719 (2462.5)	3936 (2619.2)	2330 (2530.0)
Comorbidity score, mean (SD)	2.6 (1.6)	2.5 (1.6)	2.6 (1.6)	3.2 (1.6)	3.3 (1.6)	3.9 (1.5)	1.3 (1.2)

Abbreviations: BMI, body mass index (calculated as weight in kilograms divided by height in meters squared); COVID-19, coronavirus disease 2019; ICU, intensive care unit; IQR, interquartile range; NDI, 2010 Neighborhood Socioeconomic Disadvantage Index; MM, Michigan Medicine.

^a^ Percentages are reported as fraction of column totals excluding missing entries.

^b^ Includes White Hispanic or unknown; Black Hispanic or unknown; Asian Hispanic, non-Hispanic, or unknown; Native American Hispanic, non-Hispanic, or unknown; Pacific Islander Hispanic, non-Hispanic, or unknown; and other Hispanic, non-Hispanic, or unknown.

^c^ Includes missing race and/or ethnicity.

**Figure 1.**
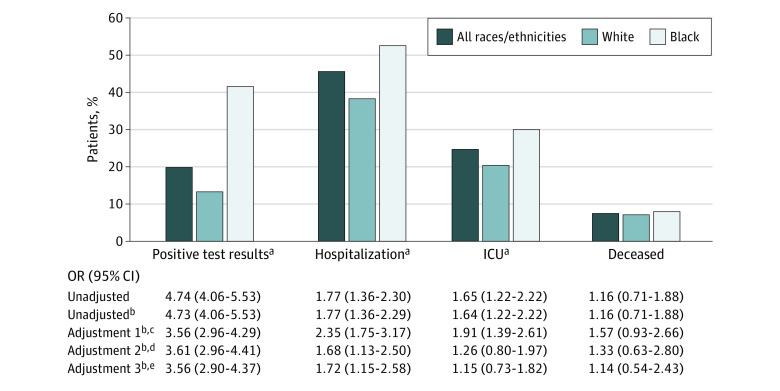
Coronavirus Disease 2019 Outcomes by Race/Ethnicity Abbreviations: ICU, intensive care unit; OR, odds ratio. ^a^χ^2^ test *P* < .001, comparing the proportion between White and Black patients. ^b^Logistic regression with Firth correction. ^c^Multivariable logistic regression with adjustment 1 (ie, age, sex, race/ethnicity; having test results positive for coronavirus disease 2019 also adjusted for population density). ^d^Multivariable logistic regression with adjustment 2 (adjustment 1 + Neighborhood Disadvantage Index). ^e^Multivariable logistic regression with adjustment 3 (adjustment 2 + comorbidity score).

Descriptive statistics stratified by White and Black patients (eTable 5 in the Supplement) suggest differences in COVID-19 outcomes across these groups (Figure 1). The test positivity rate was significantly higher among Black patients compared with White patients (442 patients [41.8%] vs 492 patients [13.2%]; *P* < .001). Similar trends were noted for hospital admission (233 patients [52.7%] vs 190 patients [38.6%]; *P* < .001) and ICU care (132 patients [29.9%] vs 101 patients [20.5%]; *P* < .001). No mortality differences by race were noted (36 patients [8.1%] vs 35 patients [7.1%]; *P* = .86).

### Association Analysis of COVID-19 Outcomes Using Multivariable Logistic Regression

#### Factors Associated With Getting Tested

Owing to limited test availability, the testing guidelines varied during the time of the study.^43^ Overall, being younger than 18 years or aged 65 to 80 years (compared with being aged 18-35 years), male sex, current smoking, and higher NDI (ie, lower socioeconomic status) were associated with a reduced chance of getting tested, whereas Black patients, patients aged 35 to 50 years (compared with age between 18 and 35 years), past smokers, individuals with overweight or obesity (ie, BMI >25), individuals who consumed alcohol, and individuals who lived in higher–population density areas or had higher comorbidity burden were associated with higher odds of getting tested (eTable 6 in the Supplement).

#### Factors Associated With Positive Test Results or With COVID-19 Susceptibility

In the full cohort, we identified several factors that differed between the COVID-19–positive group and the untested comparison group. Black patients were significantly more likely to be tested for COVID-19 and have positive test results than White patients (OR, 6.11 [95% CI, 4.83-7.73]; *P* < .001). Every 10-year increase in age was associated with increased odds of having positive test results (OR, 1.09 [95% CI, 1.05-1.14]; *P* < .001), as was higher BMI (OR per 1-unit increase, 1.03 [95% CI, 1.02-1.04]; *P* < .001), and alcohol consumption (ever vs never: OR, 1.58 [95% CI, 1.29-1.95]; *P* < .001) (Table 2). Being a current smoker (self-reported in the latest EHR encounter) was associated with a reduced chance of having positive test results (OR, 0.31 [95% CI, 0.20-0.48]; *P* < .001). In addition, residential population density was associated with positive test results (OR per 1000 persons/square mile, 1.12 [95% CI, 1.08-1.16]; *P* < .001). Having a higher comorbidity burden (OR, 1.64 [95% CI, 1.54-1.75]; *P* < .001), a respiratory condition (OR, 4.09 [95% CI, 3.36-4.97]; *P* < .001), circulatory condition (OR, 2.85 [95% CI, 2.34-3.47]; *P* < .001), type 2 diabetes (OR, 2.01 [95% CI, 1.61-2.50]; *P* < .001), kidney disease (OR, 2.82 [95% CI, 2.18-3.66]; *P* < .001), liver disease (OR, 3.33 [95% CI, 2.42-4.57]; *P* < .001), or autoimmune disease (OR, 2.44 [95% CI, 1.94-3.06]; *P* < .001) were associated with positive test results (Table 2). Conversely, a naive comparison between patients with positive test results vs individuals with negative test results showed increased likelihood of negative results for those with a higher comorbidity burden (OR, 0.90 [95% CI, 0.85-0.95]; *P* < .001), a circulatory condition (OR, 0.69 [95% CI, 0.57-0.85]; *P* < .001), any cancer (OR, 0.76 [95% CI, 0.63-0.92]; *P* = .006), or preexisting kidney disease (OR, 0.62 [95% CI, 0.50-0.77]; *P* < .001).

**Table 2.** Susceptibility and Outcomes in Full Cohort

Variable	OR (95% CI)^a^			
	COVID-19 test results		COVID-19 outcome	
	Positive vs untested	Positive vs negative	Hospitalized vs not	ICU vs not
Sample size, No.				
Comparative group	5611	3656	486	615
COVID-19 outcome group	761	761	270	141
Age, y				
Per 10-year increase	1.09 (1.05-1.14)	1.2 (1.15-1.26)	1.72 (1.53-1.93)	1.45 (1.27-1.65)
<18	0.06 (0.03-0.15)	0.13 (0.05-0.31)	5.01 (0.57-44.30)	8.75 (0.90-85.20)
18 to <35	1 [Reference]	1 [Reference]	1 [Reference]	1 [Reference]
35 to <50	1.56 (1.03-2.37)	1.23 (0.85-1.76)	0.75 (0.32-1.77)	0.72 (0.25-2.10)
50 to <65	1.59 (0.82-3.11)	2.38 (1.30-4.34)	0.72 (0.20-2.53)	0.86 (0.19-3.87)
65 to <80	1.20 (0.46-3.09)	2.53 (1.06-6.02)	0.58 (0.10-3.44)	0.71 (0.09-5.68)
≥80	1.32 (0.38-4.60)	2.94 (0.93-9.30)	1.15 (0.10-13.10)	0.64 (0.04-10.10)
Male sex	0.90 (0.76-1.07)	1.54 (1.31-1.82)	1.91 (1.36-2.68)	2.25 (1.52-3.34)
BMI				
Per 1-unit increase	1.03 (1.02-1.04)	1.03 (1.02-1.05)	1.04 (1.01-1.06)	1.03 (1.01-1.06)
<18.5	0.65 (0.27-1.54)	0.67 (0.29-1.58)	1.80 (0.24-13.60)	2.09 (0.22-20.30)
18.5 to <25	1 [Reference]	1 [Reference]	1 [Reference]	1 [Reference]
25 to <30	1.62 (1.25-2.10)	1.44 (1.13-1.83)	2.20 (1.23-3.94)	1.59 (0.79-3.21)
≥30	1.70 (1.33-2.18)	1.78 (1.42-2.24)	2.43 (1.38-4.30)	2.17 (1.10-4.26)
Smoking status				
Ever	0.73 (0.59-0.89)	0.69 (0.57-0.83)	1.11 (0.77-1.60)	1.36 (0.90-2.07)
Never	1 [Reference]	1 [Reference]	1 [Reference]	1 [Reference]
Past	0.92 (0.74-1.14)	0.89 (0.67-0.99)	1.21 (0.83-1.77)	1.47 (0.96-2.24)
Current	0.31 (0.20-0.48)	0.31 (0.20-0.47)	0.51 (0.17-1.52)	0.67 (0.17-2.64)
Alcohol consumption	1.58 (1.29-1.95)	0.95 (0.79-1.14)	0.83 (0.56-1.23)	1.10 (0.69-1.74)
Race/ethnicity				
White	1 [Reference]	1 [Reference]	1 [Reference]	1 [Reference]
Black	6.11 (4.83-7.73)	3.56 (2.90-4.37)	1.72 (1.15-2.58)	1.15 (0.73-1.82)
Other^b^	1.67 (1.26-2.23)	1.57 (1.19-2.06)	1.42 (0.79-2.54)	0.86 (0.42-1.78)
Unknown	0.11 (0.08-0.17)	0.78 (0.51-1.20)	0.72 (0.28-1.84)	0.60 (0.18-2.04)
NDI	0.04 (0.01-0.14)	0.94 (0.30-2.95)	5.51 (0.74-41.10)	13.7 (1.46-128)
Population density, per 1000 persons/square mile	1.12 (1.08-1.16)	1.07 (1.03-1.11)	1.10 (1.01-1.19)	1.08 (0.99-1.19)
Comorbidity				
Comorbidity score	1.64 (1.54-1.75)	0.90 (0.85-0.95)	1.15 (1.03-1.29)	1.16 (1.02-1.32)
Respiratory disease^c^	4.09 (3.36-4.97)	0.90 (0.73-1.09)	0.81 (0.53-1.23)	0.98 (0.60-1.61)
Circulatory disease^c^	2.85 (2.34-3.47)	0.69 (0.57-0.85)	1.35 (0.87-2.08)	1.21 (0.71-2.05)
Any cancer^c^	1.18 (0.96-1.45)	0.76 (0.63-0.92)	0.90 (0.61-1.32)	0.93 (0.60-1.44)
Type 2 diabetes^c^	2.01 (1.61-2.50)	1.07 (0.87-1.30)	1.82 (1.25-2.64)	1.50 (0.99-2.28)
Kidney disease^c^	2.82 (2.18-3.66)	0.62 (0.50-0.77)	2.87 (1.87-4.42)	2.74 (1.76-4.26)
Liver disease^c^	3.33 (2.42-4.57)	0.80 (0.62-1.04)	0.997 (0.59-1.68)	0.95 (0.52-1.73)
Autoimmune disease^c^	2.44 (1.94-3.06)	0.95 (0.78-1.15)	1.24 (0.83-1.85)	1.45 (0.92-2.29)

Abbreviations: BMI, body mass index (calculated as weight in kilograms divided by height in meters squared); ICU, intensive care unit; OR, odds ratio; NDI, Neighborhood Socioeconomic Disadvantage Index.

^a^ The model results were from Firth-corrected multivariable logistic regression *logit P*(*Y_COVID_* = *1|X, Covariate*) = *β_0_* +  *β_X_X* + *β_cov_Covariate*, in which *Y_COVID_* is the COVID-19 outcomes (ie, positive test results, hospitalization, or ICU admission); *race* includes 4 categories (ie, White, Black, other known race/ethnicity, and unknown race/ethnicity); *Covariate* = age + sex + race + NDI + comorbidity score (+ population density in susceptibility model).

^b^ Includes White Hispanic or unknown; Black Hispanic or unknown; Asian Hispanic, non-Hispanic, or unknown; Native American Hispanic, non-Hispanic, or unknown; Pacific Islander Hispanic, non-Hispanic, or unknown; and other Hispanic, non-Hispanic, or unknown.

^c^ Not adjusted for composite comorbidity score.

**Figure 2.**
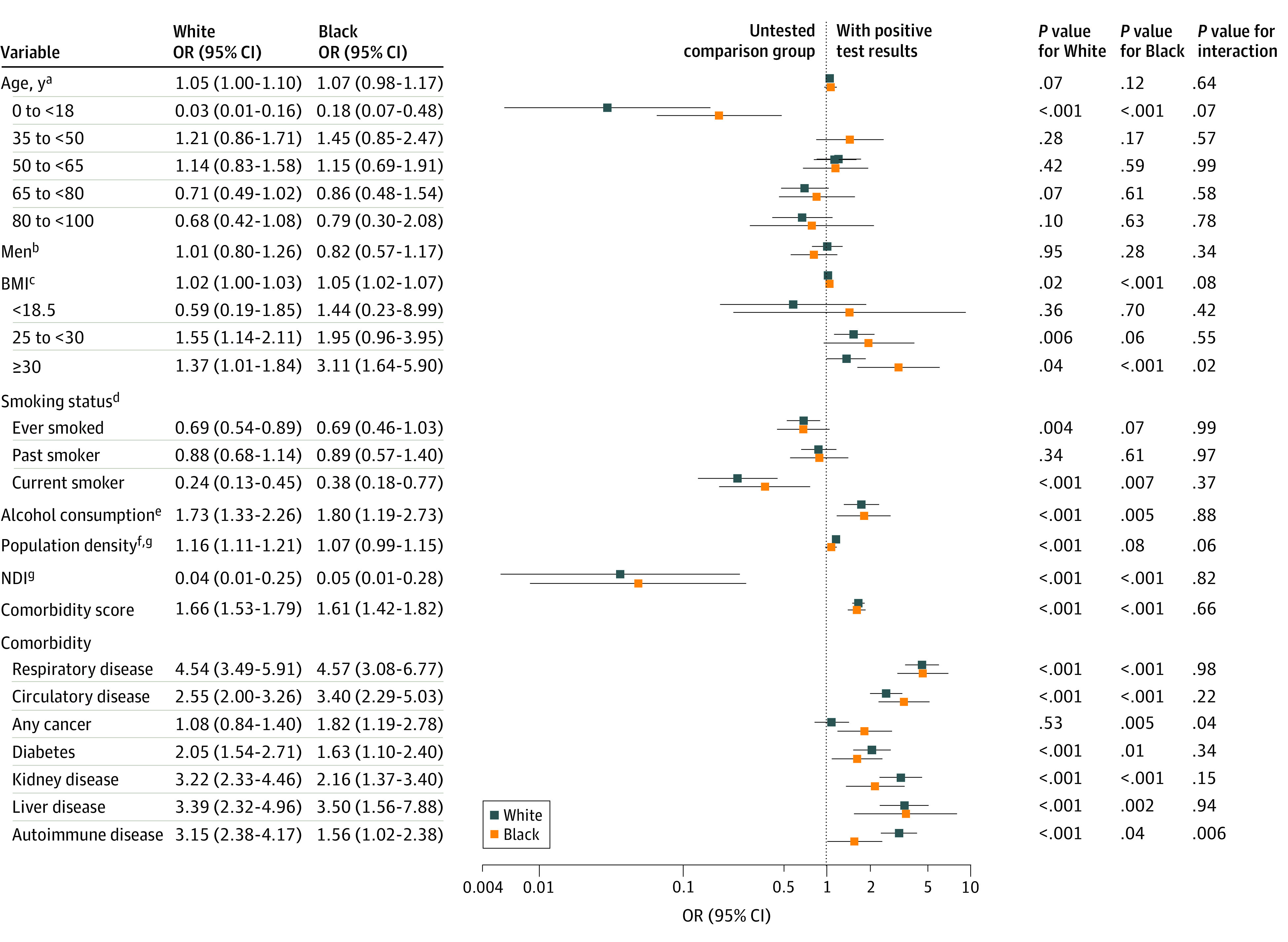
Coronavirus Disease 2019 Susceptibility White and Black Patients The results were from model *logit P*(*Y_COVID_* = *1|X*, *Covariate*) = *β_0_* + *β_X_X* + *β_Race_Race* + *β_int_X* × *Race* + *β_cov_Covariate*, in which *Covariate* = age + sex + NDI (+ comorbidity score in demographic and socioeconomic status models). ^a^Reference: age 18 to younger than 35 years. ^b^Reference: women. ^c^Reference: body mass index (BMI; calculated as weight in kilograms divided by height in meters squared) 18.5 to less than 25. ^d^Reference: never smoker. ^e^Reference: no alcohol consumption. ^f^Per 1000 persons per square mile. ^g^From 2010 census data.

Although obesity (ie, BMI >30) was associated with positive test results in both races (White: OR, 1.37 [95% CI, 1.01-1.84]; *P* = .04; Black: OR, 3.11 [95% CI, 1.64-5.90]; *P* < .001), it had stronger association in Black patients (*P_int_* = .02) (Figure 2). Having autoimmune diseases was associated with positive test results in both races (White: OR, 3.15 [95% CI, 2.38-4.17]; *P* < .001. Black: OR, 1.56 [95% CI, 1.02-2.38]; *P* = .04), but with stronger association in White patients (*P *for interaction = .006). Having any cancer was associated with positive test results in Black patients only (OR, 1.82 [95% CI, 1.19-2.78]; *P* = .005) and not in White patients (OR, 1.08 [95% CI, 0.84-1.40]; *P* = .53; *P *for interaction = .04).

#### Factors Associated With Outcomes Among Patients With COVID-19

Among the COVID-19–positive cohort, Black patients were more likely to be hospitalized (OR, 1.72 [95% CI, 1.15-2.58];*P* = .009) (Table 2; Figure 1). There was no significant difference in ICU admission by race after adjusting for covariates. Higher population density was associated with higher chance of hospitalization (OR per 1000 persons/square mile, 1.10 [95% CI, 1.01-1.19]; *P* = .02), whereas older age, male sex, and obesity were consistently associated with worse outcomes (Table 2). Type 2 diabetes (OR, 1.82 [95% CI 1.25-2.64]; *P* = .002) and kidney disease (OR, 2.87 [95% CI, 1.87-4.42]; *P* < .001) stood out as having the highest risk for hospitalization among the 7 comorbidities, and kidney disease was also associated with ICU admission (OR, 2.74 [95% CI, 1.76-4.26]; *P* < .001).

**Figure 3.**
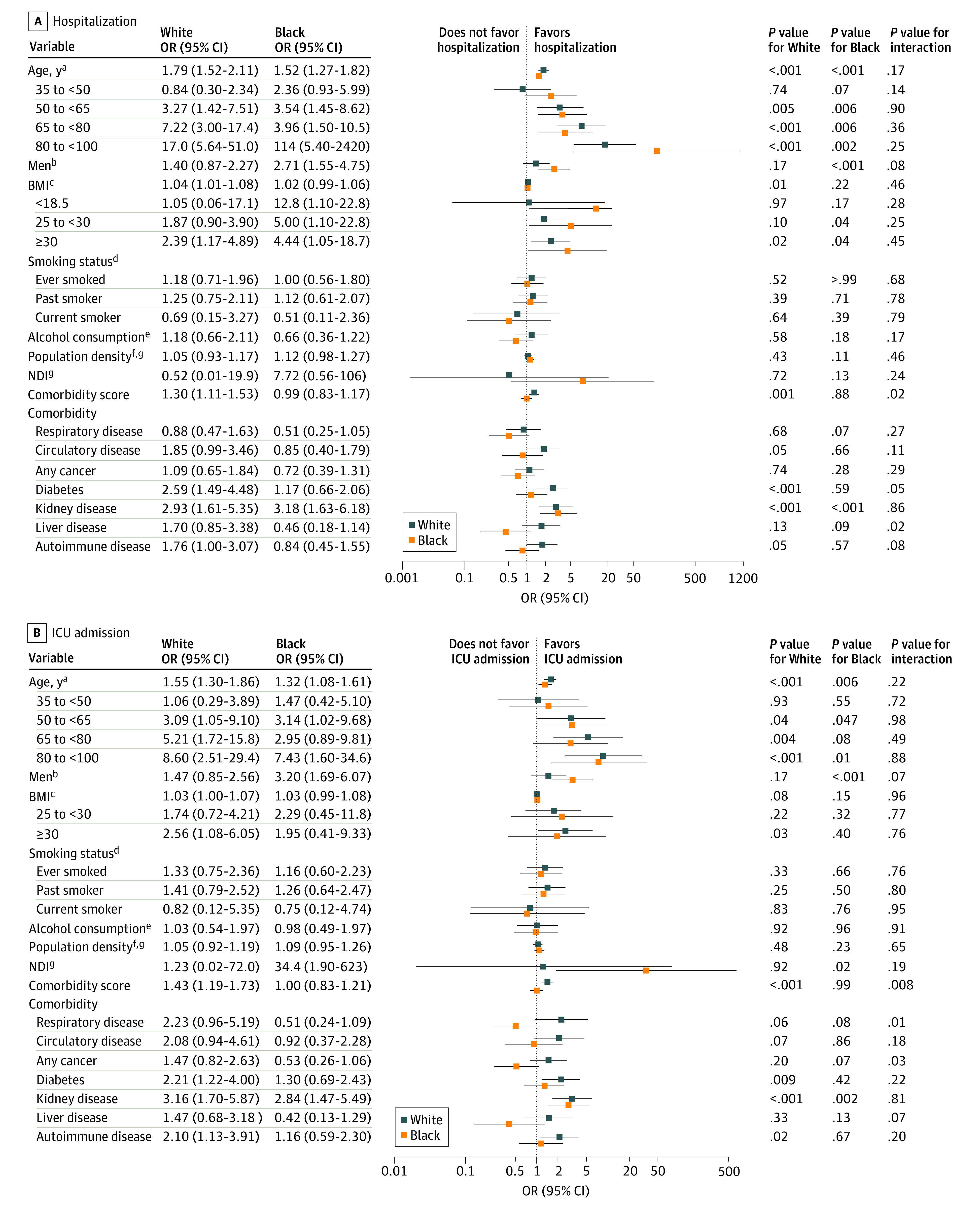
Coronavirus Disease 2019 Outcomes for White and Black Patients The results were from model *logit P*(*Y_COVID_* = *1|X, Covariate*) = *β_0_* + *β_X_X* + *β_Race_Race* + *β_int_X* × Race + *β_cov_Covariate*, in which *Y_COVID_* = *Y_hospitalization_* (A) or *Y_COVID_* = *Y_ICU_* (B) and *Covariate* = age + sex + NDI (+ comorbidity score in demographic and socioeconomic status models). ^a^Reference: age 18 to younger than 35 years. ^b^Reference: women. ^c^Reference: BMI 18.5 to less than 25. ^d^Reference: never smoker. ^e^Reference: no alcohol consumption. ^f^Per 1000 persons per square mile. ^g^From 2010 census data.

In White patients, higher comorbidity burden was associated with hospitalization (OR, 1.30 [95% CI, 1.11-1.53]; *P* = .001) and ICU admission (OR, 1.43 [95% CI, 1.19-1.73]; *P* < .001]), but not in Black patients (hospitalization: OR, 0.99 [95% CI, 0.83-1.17]; *P* = .88; *P *for interaction = .02; ICU: OR, 1.00 [95% CI, 0.83-1.21]; *P* = .99; *P *for interaction = .008) (Figure 3). Moreover, type 2 diabetes was associated with hospitalization in White patients (OR, 2.59 [95% CI, 1.49-4.48]; *P* < .001) but not in Black patients (OR, 1.17 [95% CI, 0.66-2.06; *P* = .59; *P_int_* = .046). Although no significant risk association was identified in either race, we identified a significant interaction indicating higher risk of ICU admission in White patients compared with Black patients with respiratory disease (OR, 2.23 [95% CI, 0.96-5.19]; *P* = .06 vs OR, 0.51 [95% CI, 0.24-1.09]; *P* = .08; *P *for interaction = .01) or any cancer (OR, 1.47 [95% CI, 0.82-2.63]; *P* = .20 vs OR, 0.53 [95% CI, 0.26-1.06]; *P* = .07; *P *for interaction = .03).

## Discussion

This cohort study adds to the evolving literature on using patient residential-level socioeconomic status, sociodemographic factors, and health conditions from EHR data to examine potential risk factors for observed racial disparities in COVID-19 susceptibility and prognosis. While there were significant differences in test positivity and hospitalization rates by race, there were no significant racial disparities noted in odds of poor outcomes (ie, ICU admission and mortality) after adjustment of covariates based on our data, a result consistent with Yehia et al.^52^ Higher comorbidity burden was associated with worse outcomes overall, with statistically significant differences by race. Supplementary analysis indicated that among patients with positive COVID-19 test results, Black patients had a significantly higher comorbidity score prior to COVID-19 testing compared with White patients and had a higher symptom burden at the time of getting tested (eTable 7 in the Supplement).

Testing and outcome data from integrated health systems, such as MM, in combination with neighborhood socioeconomic status data derived from the US census data are useful in quantifying risk factors for COVID-19 and associated disparities. Our association results do not explain why there are differences in COVID-19 outcomes associated with race; thus, the idea of structural factors influencing health is pivotal. This cohort study’s novel contributions include the comparison of a COVID-19 tested population with a random subset of the MM population that was not tested to avoid the biased sampling of who gets tested for COVID-19 and consideration of outcomes associated with both susceptibility and prognosis. Lastly, we present a comprehensive analytic framework that attempts to adjust for an expanded set of potential confounders with suitably chosen comparison groups, a critical need when characterizing differences in White and Black patients.

Owing to the prioritized testing protocols, there may be many asymptomatic or mildly symptomatic patients in the randomly chosen comparison group who were never tested but actually had SARS-CoV-2 infection. Therefore, the comparison results between the positive and the untested comparison group suggests that in general, individuals with preexisting health conditions had higher risk of developing severe disease outcomes after being infected with SARS-CoV-2. In contrast, a naive comparison between individuals with positive and negative results in the tested population leads to counterintuitive findings, such as a protective association of having higher comorbidity burden, a circulatory condition, any cancer or preexisting kidney disease, contradicting findings in other COVID-19 studies.^41,42^ This amplifies the need for choosing an appropriate comparison group. Alternatively, if the appropriate comparison group is not available, one can consider creating a model for who was tested and use the inverse probability weighting approach to adjust for the selection bias due to prioritized testing guidelines.^53^

In general, our findings are consistent with existing studies. Male sex was associated with a higher risk of hospitalization and death, especially among individuals 50 years and older.^54^ Similarly, health conditions, such as obesity, cancer, type 2 diabetes, and renal conditions, were prevalent among patients with worse COVID-19 outcomes.^34,45,46,55^ Notably, our findings largely agree with recent published work examining racial/ethnic differences in COVID-19 outcomes, which found Black patients had a higher hospitalization rate,^8^ increased odds of positive test results,^12^ and disproportionately high COVID-19 diagnosis rate^11^ compared with White patients. Similar directional results but different strength of association with socioeconomic status variables are likely because we used a continuous metric as opposed to the categorical measures used in Price-Haywood et al.^14^ Moreover, we also identified type 2 diabetes and kidney disease as risk factors associated hospitalization.

### Limitations

This study has several limitations. First, since this study was based on patient data at MM, we only had hospitalization records for those who were treated at MM. Thus, we may not have captured all hospitalized patients, given that only half (50.5%) of the COVID-19–positive cohort received primary care at MM. It is possible that some of the nonhospitalized patients actually were hospitalized elsewhere. Second, we did not consider the small number of transfer patients from other hospitals as a special subgroup, although they often had more severe outcomes. Third, early in the COVID-19 pandemic, all patients with COVID-19 at MM were placed in regional infectious containment units, some of whom did not require ICU-level care. We suggest future studies define ICU patients as those requiring mechanical ventilators. Fourth, one may argue that the comparison group is intrinsically different than the tested cohort and does not serve as a proper comparison group, which may impact the estimation of the ORs observed in the susceptibility models. The sensitivity analysis that restricted the patients to those who sought primary care at MM showed largely similar results (eTable 6 in the Supplement). However, this is mostly relevant for susceptibility models; the prognosis models focused on the COVID-19–positive cohort and did not use the comparison group and thus, are not subject to the same selection issues.

## Conclusions

The findings of this cohort study highlight that poor COVID-19 outcomes are disproportionately associated with at-risk populations: elderly adults, those with preexisting conditions, and those in population-dense communities. Our results support targeted screening for elderly adults and those with type 2 diabetes and kidney disease. Moreover, we call for increased investments in testing and prevention efforts in lower–socioeconomic status, densely populated, and racially diverse communities. It is these same communities that are home to a greater proportion of essential workers and thus need increased testing and protection.

## Notes

Supplement. **eFigure.** Flow Diagram of Patients Tested and Subsequent Patient Outcomes, Stratified by Race/Ethnicity **eTable 1.** Definition and Sources of All Variables and 4 Adjustments **eTable 2.** Odds Ratio of COVID-19 Outcomes From Logistic Regression for Full Cohort **eTable 3.** Comparison of Race/Ethnicity-Interaction Analysis and Race/Ethnicity-Stratified Analysis in COVID-19 Susceptibility **eTable 4.** Sensitivity Analysis Using Patients With Primary Care at MM **eTable 5.** Observed Missingness Across Variables and Descriptive Characteristics of the COVID-19 Tested or Diagnosed Cohort of the Full Cohort and White and Black Patients **eTable 6.** Odds Ratio of Susceptibility From Logistic Regression for Full Cohort **eTable 7.** Number of Key COVID-19 Symptoms within 14 days Before the First Test
