# Errors in Table, Figure Legend, and the Supplement

**DOI:** 10.1001/jamanetworkopen.2021.26218

**Published:** 2021-08-16

In the Original Investigation titled “Characteristics Associated With Racial/Ethnic Disparities in COVID-19 Outcomes in an Academic Health Care System,”^1^ published October 21, 2020, there were errors reporting the odds ratios and 95% CIs for the categorical age variable in Table 2 and eTables 2, 3, 4, and 6 in the Supplement. In addition, population density was inadvertently omitted from the covariates listed in the legend of Figure 2. This article has been corrected.^1^
