# Supplementary material for: Characteristics Associated With Racial/Ethnic Disparities in COVID-19 Outcomes in an Academic Health Care System
Source: JAMA Netw Open. 2020 Oct 21;3(10):e2025197. doi: 10.1001/jamanetworkopen.2020.25197 (PMC7578774; doi:10.1001/jamanetworkopen.2020.25197)
Supplement: Supplement. — eFigure. Flow Diagram of Patients Tested and Subsequent Patient Outcomes, Stratified by Race/Ethnicity eTable 1. Definition and Sources of All Variables and 4 Adjustments eTable 2. Odds Ratio of COVID-19 Outcomes From Logistic Regression for Full Cohort eTable 3. Comparison of Race/Ethnicity-Interaction Analysis and Race/Ethnicity-Stratified Analysis in COVID-19 Susceptibility eTable 4. Sensitivity Analysis Using Patients With Primary Care at MM eTable 5. Observed Missingness Across Variables and Descriptive Characteristics of the COVID-19 Tested or Diagnosed Cohort of the Full Cohort and White and Black Patients eTable 6. Odds Ratio of Susceptibility From Logistic Regression for Full Cohort eTable 7. Number of Key COVID-19 Symptoms within 14 days Before the First Test [file jamanetwopen-e2025197-s001.pdf]

Supplemental Online Content

Gu T, Mack JA, Salvatore M, et al. Characteristics associated with racial/ethnic disparities in COVID-19 outcomes in an academic health care system. *JAMA Netw Open*. 2020;3(10):e2025197. doi:10.1001/jamanetworkopen.2020.25197

eFigure. Flow Diagram of Patients Tested and Subsequent Patient Outcomes, Stratified by Race/Ethnicity
eTable 1. Definition and Sources of All Variables and 4 Adjustments
eTable 2. Odds Ratio of COVID-19 Outcomes From Logistic Regression for Full Cohort
eTable 3. Comparison of Race/Ethnicity-Interaction Analysis and Race/Ethnicity-Stratified Analysis in COVID-19 Susceptibility
eTable 4. Sensitivity Analysis Using Patients With Primary Care at MM
eTable 5. Observed Missingness Across Variables and Descriptive Characteristics of the COVID-19 Tested or Diagnosed Cohort of the Full Cohort and White and Black Patients
eTable 6. Odds Ratio of Susceptibility From Logistic Regression for Full Cohort
eTable 7. Number of Key COVID-19 Symptoms within 14 days Before the First Test

This supplemental material has been provided by the authors to give readers additional information about their work.

© 2020 Gu T et al. *JAMA Network Open.*

eFigure. Flow Diagram of Patients Tested and Subsequent Patient Outcomes, Stratified by Race/Ethnicity

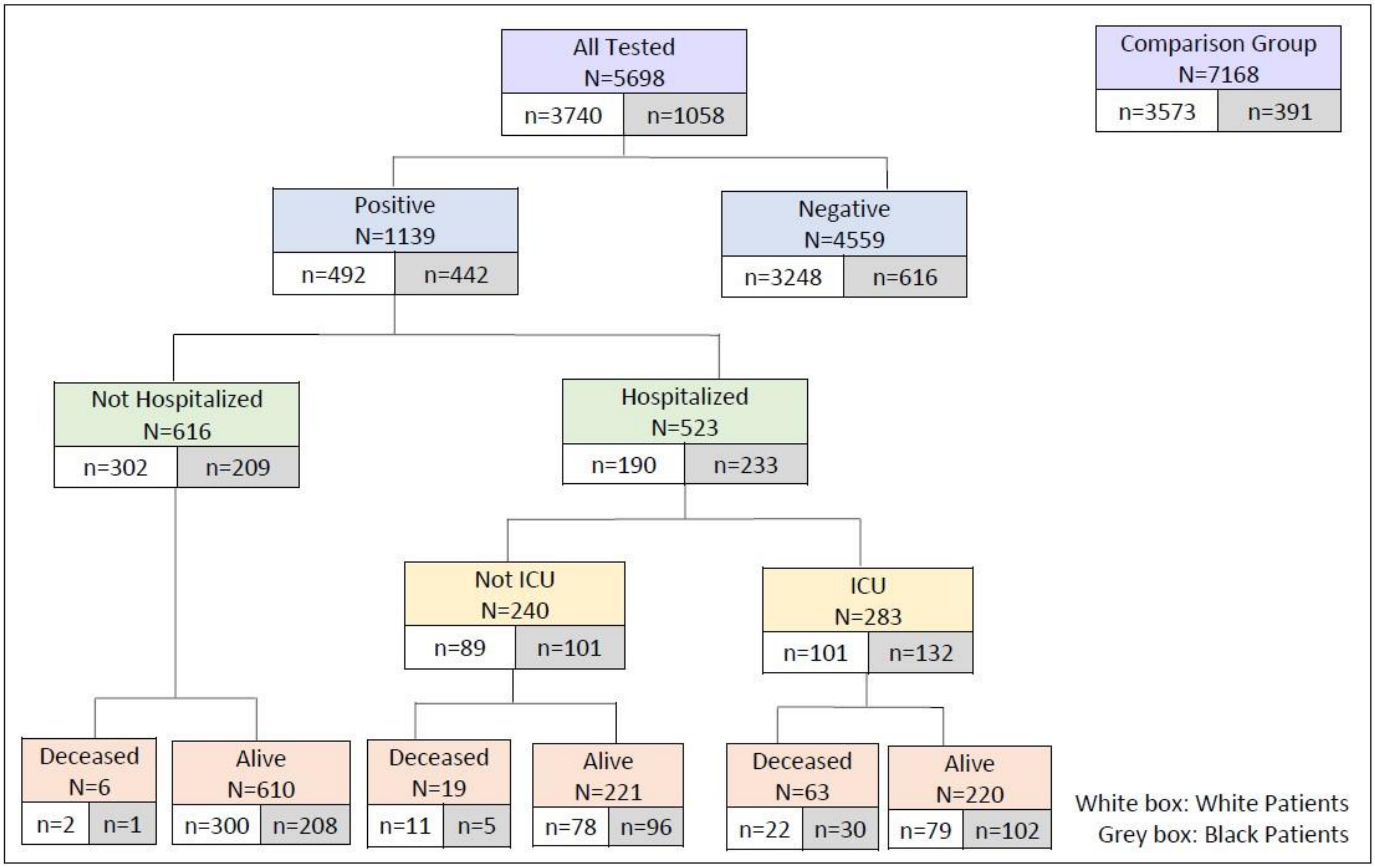

In each COVID-19 outcome, we listed the total number of patients, as well as the total number of White patients (in the white box) and Black patients (in the grey box)

© 2020 Gu T et al. *JAMA Network Open.*

eTable 1. Definition and Sources of All Variables and 4 Adjustments

**Variable**	**Definition**	**Sources**
Age	Age of patient as of the data pull: 4/22/2020	Electronic Health Record (EPIC)
Male	Gender of patient as reported.	Electronic Health Record (EPIC)
Primary Care in MM	If the patient has had an encounter in any of the primary care locations in MM since 01-01-2018, then 1; otherwise 0	Derived from the Electronic Health Record
BMI	Excluded entries if (1) age at BMI measurement was missing or below 18 years, (2) height and/or weight were missing, (3) height measurements were below 69 cm or above 234 cm, (4) weight was above 400 kg, (5) BMI deviated more than one unit from BMI calculated from height and weight (BMI = weight [in kg] / height [in m]^2). Outliers for multiple values per person were defined as values that exceeded the median BMI +/- 3 x the median absolute deviation (MAD). Final BMI values was calculated as the median BMI of the remaining entries.	Derived from the Electronic Health Record
Ever-Smoker	If the last reported smoking status is "never", but reported smoking before 1; If the last reported smoking status is "never", and never reported smoking before, then 0	Derived from the Electronic Health Record
**Smoking Status**		Self-Reported under Patient History in EHR
Never	If the patient never say that they are a 'former' smoker or 'current' smoker then 1, otherwise 0	
Past	If the last smoking status is "former", then 1	
Current	If the last smoking status is "current", then 1	
**Alcohol Consumption**	If the reported alcohol drinker status in the EHR was reported "yes" at least once; and never reported alcohol drinker before, then 0	Self-Reported under Patient History in EHR
**Race/Ethnicity**		Patient Reported - Derived from the Electronic Health Record
White	If race was reported as "Caucasian" and ethnicity as "Hispanic or Latino"	
Black	If race was reported as "African American" and ethnicity as "Hispanic or Latino"	
Other / Known Ethnicity	If race was not reported as "African American" or "Caucasian"' and ethnicity was reported as "Non-Hispanic or Latino" or "Hispanic or Latino"	
Other / Unknown Ethnicity	If race and/or ethnicity were missing	
**SES**	Data defined by US census tract (based on residential address available in each patient's EHR) for the year 2010 from the US Census and the American Community Survey (ACS).	The boundaries for the census tracts were normalized by 2010 tract boundaries using the Longitudinal Tract Data Base (Logan, Xu, and Stults, 2014).
NDI	2010 Neighborhood Socioeconomic Disadvantage Index (without Proportion Black): mean of proportion of Population in Poverty; Unemployed; with Public Assistance Income; and Female-Headed Families with children.	
Population density (1000-people/mi^2)	Population Density of the neighborhood that the patient lives in.	
**Comorbidities**	Also see Supplementary Table S7	Electronic Health Record (EPIC)

Respiratory Diseases	At least one of the following observed phecodes and their subcodes:<br>464, 465, 465.2, 465.4, 470, 471, 472, 473, 473.1, 473.3, 473.4, 474, 474.1, 474.2, 475, 475.9, 476, 477, 478, 479, 480, 480.1, 480.11, 480.12, 480.13, 480.2, 480.3, 480.5, 481, 483, 495, 495.1, 495.11, 495.2, 496, 496.1, 496.2, 496.21, 496.3, 497, 498, 499, 500, 500.1, 500.2, 501, 502, 503, 504, 504.1, 505, 506, 507, 508, 509, 509.1, 509.2, 509.3, 509.5, 509.8, 510, 510.2, 512, 512.1, 512.2, 512.3, 512.7, 512.8, 512.9, 513, 513.3, 513.31, 513.32, 513.4, 513.8, 514, 514.1, 514.2, 516, 516.1, 519, 519.1, 519.2, 519.8, 519.9	
Circulatory Diseases	At least one of the following observed phecodes and their subcodes:<br>394, 394.1, 394.2, 394.3, 394.4, 394.7, 395, 395.1, 395.2, 395.3, 395.4, 395.6, 396, 401, 401.1, 401.2, 401.21, 401.22, 401.3, 411, 411.1, 411.2, 411.3, 411.4, 411.41, 411.8, 411.9, 414, 414.2, 415, 415.1, 415.11, 415.2, 415.21, 416, 418, 418.1, 420, 420.1, 420.2, 420.21, 420.22, 420.3, 425, 425.1, 425.11, 425.12, 425.2, 425.8, 426, 426.2, 426.21, 426.22, 426.23, 426.24, 426.25, 426.3, 426.31, 426.32, 426.4, 426.7, 426.8, 426.9, 426.91, 426.92, 427, 427.1, 427.11, 427.12, 427.2, 427.21, 427.22, 427.3, 427.4, 427.41, 427.42, 427.5, 427.6, 427.61, 427.7, 427.8, 427.9, 428, 428.1, 428.2, 428.3, 428.4, 429, 429.1, 429.2, 429.3, 429.9, 430, 430.1, 430.2, 430.3, 433, 433.1, 433.11, 433.12, 433.2, 433.21, 433.3, 433.31, 433.32, 433.5, 433.6, 433.8, 440, 440.1, 440.2, 440.21, 440.22, 440.9, 441, 441.1, 441.2, 442, 442.1, 442.11, 442.2, 442.3, 442.4, 442.8, 443, 443.1, 443.7, 443.8, 443.9, 444, 444.1, 444.2, 444.5, 446, 446.1, 446.2, 446.3, 446.4, 446.5, 446.6, 446.7, 446.8, 446.9, 447, 447.1, 447.7, 448, 450, 451, 451.2, 452, 452.1, 452.2, 452.8, 453, 454, 454.1, 454.11, 455, 456, 457, 457.2, 457.3, 458, 458.1, 458.2, 458.9, 459, 459.1, 459.7, 459.9	
Any Cancer	At least one of the following observed phecodes and their subcodes:<br>145, 145.2, 145.3, 145.4, 149, 149.1, 149.2, 149.3, 149.4, 149.5, 149.9, 150, 151, 153, 153.2, 153.3, 155, 155.1, 157, 158, 159, 159.2, 159.3, 159.4, 164, 165, 165.1, 170, 170.1, 170.2, 172, 172.1, 172.11, 172.2, 172.21, 172.22, 172.3, 174, 174.1, 174.11, 175, 180, 180.1, 180.3, 182, 184, 184.1, 184.11, 184.2, 185, 187, 187.1, 187.2, 189, 189.1, 189.11, 189.12, 189.2, 189.21, 189.4, 190, 191, 191.1, 191.11, 193, 194, 195, 195.1, 195.3, 196, 197, 198, 198.1, 198.2, 198.3, 198.4, 198.5, 198.6, 198.7, 199.4, 200, 200.1, 201, 202, 202.2, 202.21, 202.22, 202.23, 202.24, 204, 204.1, 204.11, 204.12, 204.2, 204.21, 204.22, 204.3, 204.4, 209	
Type 2 Diabetes	At least one of the following observed phecode and their subcodes: 250.2	
Kidney Diseases	At least one of the following observed phecodes and their subcodes: 585	
Liver Diseases	At least one of the following observed phecodes and their subcodes: 571	
Autoimmune Diseases	At least one of the following observed phecodes and their subcodes: 242.1, 250.1, 335, 557.1, 694.1, 695.4, 696.4, 697, 704.1, 714.1, 717	
Comorbidity Score	The summation of 7 comorbidities values above, ranging from 0 to 7	
**COVID-19 Outcomes**		Derived from RDW's COVID Registry
COVID-19 Tested	Patients who were tested for COVID-19 at the time of data pull.	
COVID-19 Positive	Patients who tested positive at least once for COVID-19	

COVID-19 Negative	Patients who always tested negative for COVID-19	
Not-Hospitalized	Patients in the positive COVID cohort who have no inpatient stays after 3/5/2020	
Hospitalized	Patients in the positive COVID cohort who checked in as an inpatient after 3/5/2020 at least once.	
ICU	Patients in the positive COVID cohort who checked into the ICU during their inpatient stay after 3/5/2020.	
Deceased	Patients in the cohort who have died based on their Electronic Health Record.	Electronic Health Record (EPIC)
**Comparison Groups**		
Unmatched Comparison Group	Randomly picked cohort of patients who are not part of the COVID-19 cohort (Tested, Positive, Negative), who are alive and who have had an encounter in MM (Inpatient, Outpatient or Emergency) since 2012-04-23. We created an untested comparison group (n=7,168) from the MM database, which is a similar-sized random sample of contemporaneous patients. Specifically, we initially extracted 20,000 individuals before limiting the group to patients who (1) were alive at the time of data pull, (2) have had encounters after 2012-04-22 and (3) who had inpatient, outpatient and/or emergency visits. At the time of the last update for COVID-19 outcomes (July 28, 2020), all patients in the comparison group were alive, and we further excluded 43 patients who were tested after the initial data extraction (April 22, 2020)	Derived from the Electronic Health Record
Matched Comparison Group	Randomly picked cohort of patients matched by Age (<=50, >50), Race/Ethnicity, Gender to be 3 times the COVID cohort for each group. Initially extracted 17,094 individuals by matching on race (White/non-Hispanic or Black), age, sex. Reduction in sample size happened as Ethnicity variable was missing in many matched Black patients, reducing the usable sample size for the matched comparison group to 13,351	
**Adjustments**		
Adjustment 0	Unadjusted	
Adjustment 1	age + sex + race/ethnicity (+ population density)*	
Adjustment 2	adjustment 1 + NDI	
Adjustment 3	adjustment 2 + comorbidity score	

Abbreviations: MM, Michigan Medicine; ICU, intensive care unit; BMI, body mass index; SES, social economics status; NDI, 2010 Neighborhood Socioeconomic Disadvantage Index.
*The population density is used only in the tested positive/susceptibility model as a covariate.

eTable 2. Odds Ratio of COVID-19 Outcomes From Logistic Regression for Full Cohort

Positive (1) vs Comparison Group (0)		Unadjusted (n_0=7165, n_1=1139)	Adjustment 1 (n_0=5910, n_1=804)	Adjustment 2 (n_0=5909, n_1=804)	Adjustment 3 (n_0=5611, n_1=761)
**Variable**		**OR (95% CI)**			
**Age (unit: 10-year)**		1.20 (1.17, 1.24)	1.26 (1.21, 1.31)	1.26 (1.21, 1.31)	1.09 (1.05, 1.14)
**Age Range** REF: [18,35)	[0,18)	0.06 (0.03, 0.12)	0.05 (0.02, 0.13)	0.06 (0.02, 0.13)	0.06 (0.03, 0.15)
	[35,50)	1.78 (1.46, 2.18)	1.80 (1.22, 2.66)	1.76 (1.19, 2.61)	1.56 (1.03, 2.37)
	[50,65)	2.02 (1.68, 2.44)	2.37 (1.26, 4.45)	2.28 (1.21, 4.28)	1.59 (0.82, 3.11)
	[65,80)	1.58 (1.29, 1.93)	2.14 (0.88, 5.22)	2.01 (0.82, 4.91)	1.20 (0.46, 3.09)
	[80,100)	1.59 (1.21, 2.08)	2.37 (0.73, 7.63)	2.17 (0.67, 7.04)	1.32 (0.38, 4.60)
**Male Sex**		1.05 (0.92, 1.19)	0.91 (0.77, 1.07)	0.91 (0.77, 1.07)	0.90 (0.76, 1.07)
**BMI**		1.06 (1.05, 1.07)	1.04 (1.03, 1.05)	1.04 (1.03, 1.05)	1.03 (1.02, 1.04)
**BMI Range** REF: [18.5,25)	<18.5	0.84 (0.42, 1.68)	0.63 (0.27, 1.49)	0.63 (0.27, 1.48)	0.65 (0.27, 1.54)
	[25,30)	1.99 (1.63, 2.44)	1.69 (1.32, 2.16)	1.70 (1.33, 2.18)	1.62 (1.25, 2.10)
	>=30	3.02 (2.51, 3.64)	1.98 (1.57, 2.51)	2.07 (1.63, 2.62)	1.70 (1.33, 2.18)
**Ever-Smoker**		1.12 (0.97, 1.30)	0.81 (0.67, 0.97)	0.84 (0.69, 1.01)	0.73 (0.59, 0.89)
**Smoking Status** REF: Never-Smoker	Past-Smoker	1.50 (1.29, 1.76)	1.09 (0.89, 1.34)	1.11 (0.90, 1.36)	0.92 (0.74, 1.14)
	Current-Smoker	0.40 (0.28, 0.55)	0.28 (0.18, 0.42)	0.30 (0.20, 0.46)	0.31 (0.20, 0.48)
**Alcohol Consumption**		1.44 (1.22, 1.69)	1.52 (1.25, 1.84)	1.46 (1.21, 1.78)	1.58 (1.29, 1.95)
**Race/Ethnicity** REF: White	Black	8.20 (6.95, 9.68)	6.08 (4.98, 7.41)	7.55 (6.06, 9.40)	6.11 (4.83, 7.73)
	Other / Known Ethnicity	1.66 (1.33, 2.06)	1.71 (1.31, 2.22)	1.68 (1.29, 2.19)	1.67 (1.26, 2.23)
	Other / Unknown Ethnicity	0.23 (0.18, 0.29)	0.09 (0.06, 0.14)	0.095 (0.06, 0.14)	0.11 (0.08, 0.17)
**SES**	Population density (1000-people/mi^2)	1.11 (1.09, 1.14)	1.08 (1.05, 1.11)	1.11 (1.08, 1.15)	1.12 (1.08, 1.16)
	NDI	9.75 (4.20, 22.7)	0.06 (0.02, 0.18)	0.06 (0.02, 0.18)	0.04 (0.02, 0.14)
**Comorbidity Score**		1.89 (1.80, 1.99)	1.63 (1.53, 1.74)	1.64 (1.54, 1.75)	1.64 (1.54, 1.75)
**Comorbidities**	Respiratory	5.26 (4.42, 6.27)	4.15 (3.41, 5.05)	4.09 (3.36, 4.97)	2.28 (1.79, 2.90)
	Circulatory	4.55 (3.86, 5.35)	2.82 (2.32, 3.43)	2.85 (2.34, 3.47)	1.23 (0.96, 1.57)
	Any Cancer	1.47 (1.25, 1.73)	1.19 (0.97, 1.46)	1.18 (0.96, 1.45)	0.52 (0.41, 0.66)
	Type 2 Diabetes	4.19 (3.53, 4.96)	1.95 (1.57, 2.43)	2.01 (1.61, 2.50)	0.72 (0.55, 0.93)
	Kidney	5.98 (4.92, 7.25)	2.71 (2.10, 3.50)	2.82 (2.18, 3.66)	0.87 (0.64, 1.19)
	Liver	3.91 (3.01, 5.08)	3.18 (2.32, 4.35)	3.33 (2.42, 4.57)	1.19 (0.83, 1.70)
	Autoimmune	3.18 (2.64, 3.84)	2.42 (1.93, 3.03)	2.44 (1.94, 3.06)	0.94 (0.72, 1.23)
**Hospitalization (1) vs Not (0)**		**Unadjusted (n_0=560, n_1=523)**	**Adjustment 1 (n_0=560, n_1=523)**	**Adjustment 2 (n_0=516, n_1=283)**	**Adjustment 3 (n_0=486, n_1=270)**

**Age (unit: 10-year)**		1.69 (1.56, 1.84)	1.71 (1.57, 1.86)	1.82 (1.64, 2.03)	1.72 (1.53, 1.93)
**Age Range** REF: [18,35)	[0,18)	3.52 (0.90, 13.7)	10.8 (2.30, 51.0)	5.28 (0.60, 46.2)	5.01 (0.57, 44.3)
	[35,50)	2.06 (1.33, 3.19)	0.63 (0.33, 1.19)	0.79 (0.34, 1.81)	0.75 (0.32, 1.77)
	[50,65)	4.13 (2.74, 6.22)	0.45 (0.17, 1.19)	0.87 (0.25, 2.97)	0.72 (0.20, 2.53)
	[65,80)	8.68 (5.56, 13.6)	0.33 (0.08, 1.29)	0.71 (0.12, 4.08)	0.58 (0.10, 3.44)
	[80,100)	25.3 (12.6, 50.8)	0.39 (0.06, 2.53)	1.30 (0.12, 13.9)	1.15 (0.10, 13.1)
**Male Sex**		2.05 (1.61, 2.59)	2.03 (1.56, 2.65)	2.09 (1.50, 2.90)	1.91 (1.36, 2.68)
**BMI**		1.02 (1.01, 1.04)	1.04 (1.02, 1.06)	1.04 (1.02, 1.06)	1.04 (1.01, 1.06)
**BMI Range** REF: [18.5,25)	<18.5	2.15 (0.56, 8.27)	1.22 (0.23, 6.42)	1.48 (0.19, 11.8)	1.80 (0.24, 13.6)
	[25,30)	1.83 (1.26, 2.68)	1.51 (0.97, 2.36)	2.21 (1.25, 3.91)	2.20 (1.23, 3.94)
	>=30	1.71 (1.20, 2.42)	1.83 (1.20, 2.79)	2.59 (1.49, 4.51)	2.43 (1.38, 4.30)
**Ever-Smoker**		1.45 (1.11, 1.90)	1.01 (0.74, 1.37)	1.25 (0.88, 1.78)	1.11 (0.77, 1.60)
**Smoking Status** REF: Never-Smoker	Past-Smoker	1.69 (1.27, 2.24)	1.11 (0.81, 1.54)	1.36 (0.94, 1.96)	1.21 (0.83, 1.77)
	Current-Smoker	0.44 (0.21, 0.94)	0.51 (0.23, 1.12)	0.62 (0.23, 1.71)	0.51 (0.17, 1.52)
**Alcohol Consumption**		0.84 (0.61, 1.14)	0.99 (0.69, 1.41)	0.81 (0.55, 1.19)	0.83 (0.56, 1.23)
**Race/Ethnicity** REF: White	Black	1.77 (1.36, 2.29)	2.35 (1.75, 3.17)	1.68 (1.13, 2.5)	1.72 (1.15, 2.58)
	Other / Known Ethnicity	1.15 (0.77, 1.73)	1.50 (0.96, 2.34)	1.32 (0.76, 2.29)	1.42 (0.79, 2.54)
	Other / Unknown Ethnicity	2.40 (1.49, 3.89)	2.07 (1.22, 3.49)	0.52 (0.20, 1.33)	0.72 (0.28, 1.84)
**SES**	Population density (1000-people/mi^2)	1.13 (1.06, 1.21)	1.12 (1.04, 1.21)	1.11 (1.02, 1.2)	1.10 (1.01, 1.19)
	NDI	6.23 (1.30, 30.0)	7.34 (1.03, 52.4)	7.34 (1.03, 52.4)	5.51 (0.74, 41.1)
**Comorbidity Score**		1.47 (1.33, 1.61)	1.21 (1.09, 1.35)	1.15 (1.03, 1.29)	1.15 (1.03, 1.29)
**Comorbidities**	Respiratory	1.20 (0.85, 1.70)	1.04 (0.70, 1.55)	0.81 (0.53, 1.23)	0.46 (0.27, 0.78)
	Circulatory	3.01 (2.09, 4.32)	1.68 (1.12, 2.52)	1.35 (0.87, 2.08)	0.98 (0.58, 1.67)
	Any Cancer	1.36 (1.00, 1.85)	0.82 (0.57, 1.18)	0.90 (0.61, 1.32)	0.62 (0.39, 0.97)
	Type 2 Diabetes	3.53 (2.60, 4.80)	1.88 (1.33, 2.65)	1.82 (1.25, 2.64)	1.63 (1.04, 2.54)
	Kidney	6.74 (4.72, 9.63)	3.72 (2.52, 5.48)	2.87 (1.87, 4.42)	3.14 (1.84, 5.38)
	Liver	1.24 (0.79, 1.93)	1.02 (0.62, 1.66)	0.997 (0.59, 1.68)	0.71 (0.40, 1.27)
	Autoimmune	1.12 (0.80, 1.57)	1.10 (0.75, 1.60)	1.24 (0.83, 1.85)	0.94 (0.58, 1.51)
**ICU (1) vs Not (0)**		**Unadjusted (n_0=850, n_1=283)**	**Adjustment 1 (n_0=850, n_1=283)**	**Adjustment 2 (n_0=652, n_1=147)**	**Adjustment 3 (n_0=615, n_1=141)**
**Age (unit: 10-year)**		1.37 (1.26, 1.49)	1.36 (1.25, 1.48)	1.53 (1.36, 1.72)	1.45 (1.27, 1.65)
**Age Range** REF: [18,35)	[0,18)	3.61 (0.86, 15.1)	7.28 (1.45, 36.5)	8.43 (0.88, 81.0)	8.75 (0.90, 85.2)
	[35,50)	1.10 (0.64, 1.89)	0.45 (0.21, 0.93)	0.75 (0.26, 2.11)	0.72 (0.25, 2.1)
	[50,65)	2.55 (1.59, 4.09)	0.48 (0.16, 1.40)	0.99 (0.23, 4.29)	0.86 (0.19, 3.87)
	[65,80)	4.39 (2.7, 7.15)	0.37 (0.08, 1.65)	0.86 (0.11, 6.63)	0.71 (0.09, 5.68)

	[80,100)	3.87 (2.12, 7.07)	0.17 (0.02, 1.24)	0.75 (0.05, 11.3)	0.64 (0.04, 10.1)
**Male Sex**		2.01 (1.53, 2.65)	1.97 (1.49, 2.63)	2.35 (1.60, 3.45)	2.25 (1.52, 3.34)
**BMI**		1.03 (1.01, 1.04)	1.04 (1.02, 1.05)	1.04 (1.01, 1.07)	1.03 (1.01, 1.06)
**BMI Range** REF: [18.5,25)	<18.5	1.22 (0.26, 5.77)	0.65 (0.12, 3.59)	1.55 (0.16, 15.4)	2.09 (0.22, 20.3)
	[25,30)	1.25 (0.81, 1.95)	0.99 (0.62, 1.58)	1.42 (0.72, 2.79)	1.59 (0.79, 3.21)
	>=30	1.45 (0.97, 2.18)	1.39 (0.89, 2.16)	2.17 (1.13, 4.16)	2.17 (1.10, 4.26)
**Ever-Smoker**		1.80 (1.31, 2.46)	1.33 (0.95, 1.86)	1.46 (0.97, 2.18)	1.36 (0.90, 2.07)
**Smoking Status** REF: Never-Smoker	Past-Smoker	2.02 (1.46, 2.80)	1.45 (1.02, 2.05)	1.58 (1.05, 2.39)	1.47 (0.96, 2.24)
	Current-Smoker	0.58 (0.21, 1.60)	0.64 (0.23, 1.79)	0.62 (0.16, 2.43)	0.67 (0.17, 2.64)
**Alcohol Consumption**		1.03 (0.70, 1.52)	1.07 (0.70, 1.63)	1.06 (0.67, 1.66)	1.10 (0.69, 1.74)
**Race/Ethnicity** REF: White	Black	1.64 (1.22, 2.22)	1.90 (1.39, 2.61)	1.25 (0.80, 1.96)	1.15 (0.73, 1.82)
	Other / Known Ethnicity	0.73 (0.43, 1.24)	0.84 (0.48, 1.44)	0.84 (0.42, 1.67)	0.86 (0.42, 1.78)
	Other / Unknown Ethnicity	2.39 (1.45, 3.94)	2.22 (1.32, 3.72)	0.47 (0.14, 1.58)	0.60 (0.18, 2.04)
**SES**	Population density (1000-people/mi^2)	1.13 (1.05, 1.21)	1.11 (1.02, 1.2)	1.07 (0.98, 1.17)	1.08 (0.99, 1.19)
	NDI	14.0 (2.27, 85.9)	19.7 (2.24, 173)	19.7 (2.24, 173)	13.7 (1.46, 128)
**Comorbidity Score**		1.42 (1.28, 1.58)	1.25 (1.11, 1.41)	1.16 (1.02, 1.32)	1.16 (1.02, 1.32)
**Comorbidities**	Respiratory	1.32 (0.85, 2.04)	1.23 (0.77, 1.95)	0.98 (0.60, 1.61)	0.59 (0.32, 1.08)
	Circulatory	2.56 (1.61, 4.08)	1.61 (0.98, 2.64)	1.21 (0.71, 2.05)	0.80 (0.42, 1.52)
	Any Cancer	1.17 (0.81, 1.67)	0.82 (0.55, 1.22)	0.93 (0.60, 1.44)	0.64 (0.39, 1.07)
	Type 2 Diabetes	2.68 (1.91, 3.76)	1.65 (1.14, 2.38)	1.50 (0.99, 2.28)	1.22 (0.74, 2.00)
	Kidney	5.85 (4.10, 8.34)	3.77 (2.56, 5.53)	2.74 (1.76, 4.26)	3.05 (1.72, 5.43)
	Liver	1.29 (0.77, 2.15)	1.13 (0.66, 1.93)	0.95 (0.52, 1.73)	0.66 (0.34, 1.28)
	Autoimmune	1.29 (0.87, 1.89)	1.35 (0.89, 2.05)	1.45 (0.92, 2.29)	1.14 (0.67, 1.94)
**Deceased (1) vs Alive (0)**		**Unadjusted (n_0=1051, n_1=88)**	**Adjustment 1 (n_0=1051, n_1=88)**	**Adjustment 2 (n_0=756, n_1=48)**	**Adjustment 3 (n_0=714, n_1=47)**
**Age (unit: 10-year)**		2.21 (1.87, 2.62)	2.23 (1.87, 2.66)	2.49 (1.94, 3.2)	2.31 (1.78, 3)
**Age Range** REF: [18,35)	[0,18)	0 (0, 0)	0 (0, 0)	0 (0, 0)	0 (0, 0)
	[35,50)	1.76 (0.39, 7.95)	0.78 (0.14, 4.38)	1.22 (0.13, 12)	1.10 (0.11, 10.6)
	[50,65)	3.99 (1.04, 15.3)	0.89 (0.10, 7.64)	2.04 (0.12, 36.2)	1.51 (0.09, 26.6)
	[65,80)	12.5 (3.40, 46.3)	1.32 (0.08, 22.0)	6.26 (0.14, 272)	4.38 (0.10, 187)
	[80,100)	51.0 (13.6, 191)	3.35 (0.10, 117)	31.9 (0.24, 4190)	23.0 (0.18, 300)
**Male Sex**		2.11 (1.35, 3.30)	2.12 (1.31, 3.45)	3.25 (1.65, 6.38)	3.05 (1.55, 6.02)
**BMI**		0.997 (0.97, 1.02)	1.02 (0.998, 1.04)	1.06 (1.01, 1.11)	1.05 (1.00, 1.10)
**BMI Range** REF: [18.5,25)	<18.5	2.12 (0.31, 14.4)	0.63 (0.07, 5.78)	3.74 (0.21, 66.6)	6.76 (0.39, 118)
	[25,30)	1.18 (0.60, 2.32)	1.03 (0.49, 2.18)	0.91 (0.30, 2.79)	0.88 (0.28, 2.71)
	>=30	1.01 (0.54, 1.92)	1.58 (0.76, 3.28)	2.31 (0.79, 6.75)	2.12 (0.73, 6.20)

**Ever-Smoker**		3.8 (2.14, 6.74)	2.00 (1.08, 3.71)	1.72 (0.86, 3.42)	1.49 (0.74, 3.00)
**Smoking Status** REF: Never-Smoker	Past-Smoker	4.39 (2.47, 7.81)	2.12 (1.14, 3.94)	1.81 (0.90, 3.61)	1.56 (0.77, 3.15)
	Current-Smoker	0.402 (0.02, 7.03)	0.77 (0.04, 13.5)	1.06 (0.06, 19.5)	1.03 (0.05, 21.1)
**Alcohol Consumption**		1.02 (0.53, 1.96)	0.97 (0.47, 2.01)	1.16 (0.54, 2.48)	1.26 (0.58, 2.72)
**Race/Ethnicity** REF: White	Black	1.16 (0.72, 1.87)	1.57 (0.93, 2.66)	1.33 (0.63, 2.8)	1.14 (0.54, 2.43)
	Other / Known Ethnicity	0.38 (0.12, 1.17)	0.54 (0.17, 1.73)	0.80 (0.23, 2.77)	0.81 (0.23, 2.86)
	Other / Unknown Ethnicity	2.65 (1.37, 5.15)	2.26 (1.10, 4.67)	1.29 (0.35, 4.76)	2.00 (0.52, 7.70)
**SES**	Population density (1000-people/mi^2)	1.15 (1.03, 1.29)	1.1 (0.97, 1.25)	1.06 (0.925, 1.22)	1.07 (0.93, 1.24)
	NDI	8.03 (0.462, 140)	23.4 (0.65, 849)	23.4 (0.646, 849)	17.4 (0.44, 686)
**Comorbidity Score**		1.69 (1.43, 1.99)	1.48 (1.21, 1.79)	1.27 (1.03, 1.57)	1.27 (1.03, 1.57)
**Comorbidities**	Respiratory	1.65 (0.78, 3.46)	1.72 (0.76, 3.86)	1.15 (0.50, 2.64)	0.51 (0.18, 1.45)
	Circulatory	4.61 (1.74, 12.2)	2.34 (0.82, 6.70)	1.52 (0.53, 4.38)	0.75 (0.21, 2.64)
	Any Cancer	2.28 (1.37, 3.81)	1.43 (0.80, 2.57)	1.59 (0.83, 3.07)	1.13 (0.53, 2.40)
	Type 2 Diabetes	6.22 (3.63, 10.7)	3.72 (2.06, 6.70)	2.28 (1.19, 4.37)	1.79 (0.83, 3.88)
	Kidney	8.01 (4.67, 13.7)	3.94 (2.17, 7.13)	2.85 (1.46, 5.57)	2.55 (1.06, 6.18)
	Liver	1.33 (0.62, 2.84)	1.35 (0.59, 3.07)	0.90 (0.34, 2.42)	0.54 (0.19, 1.54)
	Autoimmune	0.72 (0.36, 1.43)	0.81 (0.38, 1.69)	0.84 (0.38, 1.86)	0.43 (0.17, 1.07)

Abbreviations: OR, odds ratio; ICU, intensive care unit; BMI, body mass index; NA, not applicable; REF, reference group; SES, social economics status; NDI, 2010 Neighborhood Socioeconomic Disadvantage Index; adjustment 0, unadjusted; adjustment 1, age+sex+race/ethnicity+(persons per mile^2 in susceptibility model only); adjustment 2, adjustment 1+NDI; adjustment 3, adjustment 2+comorbidity score.

The model used was: $\text{logit } P(Y_{COVID} = 1 | X, \text{adjustment}) = \beta_0 + \beta_X X + \beta_{adjust}\text{adjustment}_j$. Here Y_{COVID} is various COVID-19 related outcomes under consideration (i.e., COVID-19 positive, hospitalization and ICU admission); X is the variable/risk factor of interest; and adjustment_j, j = 0, …,3 are the four nested covariate adjustment models listed in eTable 1.

eTable 3. Comparison of Race/Ethnicity-Interaction Analysis and Race/Ethnicity-Stratified Analysis in COVID-19 Susceptibility

**Positive (1) vs Comparison Group* (0)**		**White Patients**				**Black Patients**				**White vs Black Patients**	
		**OR**		**P-value**		**OR**		**P-value**		**P-value**	
**Variables**		**Interaction**	**Stratified**	**Interaction**	**Stratified**	**Interaction**	**Stratified**	**Interaction**	**Stratified**	**Interaction**	**Stratified**
**Age (unit: 10-year)**		1.05 (0.996, 1.1)	0.97 (0.92, 1.02)	0.073	0.243	1.07 (0.98, 1.17)	1.06 (0.98, 1.14)	0.115	0.176	0.636	0.076
**Age Range** REF: [18,35)	[0,18)	0.03 (0.006, 0.16)	0.036 (0.007, 0.18)	**2.67E-05**	**5.51E-05**	0.18 (0.07, 0.48)	0.16 (0.06, 0.40)	**6.41E-04**	**8.33E-05**	0.066	0.114
	[35,50)	1.21 (0.86, 1.71)	1.2 (0.86, 1.68)	0.282	0.282	1.45 (0.85, 2.47)	1.47 (0.94, 2.32)	0.171	0.0945	0.573	0.482
	[50,65)	1.14 (0.83, 1.58)	0.998 (0.72, 1.38)	0.417	0.99	1.15 (0.69, 1.91)	1.13 (0.72, 1.77)	0.589	0.599	0.986	0.664
	[65,80)	0.71 (0.49, 1.02)	0.56 (0.39, 0.80)	0.066	**0.00171**	0.86 (0.48, 1.54)	0.79 (0.47, 1.33)	0.61	0.37	0.58	0.287
	[80,100)	0.68 (0.42, 1.08)	0.46 (0.29, 0.73)	0.105	**0.00091**	0.79 (0.30, 2.08)	0.65 (0.3, 1.43)	0.633	0.285	0.78	0.460
**Male Sex**		1.01 (0.81, 1.26)	1.24 (1.01, 1.54)	0.951	**0.0434**	0.82 (0.57, 1.17)	1.23 (0.90, 1.68)	0.275	0.187	0.337	0.960
**BMI**		1.02 (1, 1.03)	1.02 (1, 1.03)	**1.76E-02**	**0.0063**	1.05 (1.02, 1.07)	1.02 (1.00, 1.03)	**5.67E-04**	**0.0462**	0.079	0.808
**BMI Range** REF: [18.5,25)	<18.5	0.59 (0.19, 1.9)	0.69 (0.23, 2.09)	0.365	0.5120	1.44 (0.23, 8.99)	1.32 (0.29, 6.06)	0.696	0.72	0.418	0.500
	[25,30)	1.55 (1.14, 2.11)	1.44 (1.07, 1.92)	**5.71E-03**	**0.0144**	1.95 (0.97, 3.95)	2.53 (1.38, 4.63)	0.063	0.0027	0.553	0.099
	>=30	1.37 (1.01, 1.84)	1.47 (1.1, 1.95)	**0.041**	**0.0085**	3.11 (1.64, 5.9)	4.30 (2.47, 7.47)	**5.15E-04**	**2.46E-07**	**0.022**	**0.001**
**Ever-Smoker**		0.69 (0.54, 0.89)	0.711 (0.56, 0.9)	**3.81E-03**	**0.0043**	0.69 (0.46, 1.03)	0.70 (0.49, 0.99)	0.069	**0.0408**	0.99	0.930
**Smoking status** REF: Never-Smoker	Past-Smoker	0.88 (0.68, 1.14)	0.86 (0.67, 1.09)	0.337	0.21	0.89 (0.57, 1.4)	0.89 (0.61, 1.30)	0.608	0.543	0.967	0.865
	Current-Smoker	0.25 (0.13, 0.45)	0.27 (0.15, 0.50)	**7.92E-06**	**2.93E-05**	0.377 (0.19, 0.77)	0.35 (0.18, 0.671)	**0.007**	**0.00157**	0.37	0.591
**Alcohol Consumption**		1.73 (1.33, 2.26)	1.75 (1.36, 2.24)	**5.12E-05**	**1.32E-05**	1.8 (1.19, 2.73)	2.05 (1.45, 2.91)	**0.005**	**5.46E-05**	0.879	0.462
**SES**	Population density (1000-people/mi^2)	1.16 (1.11, 1.21)	1.13 (1.09, 1.18)	**1.24E-10**	**2.24E-10**	1.07 (0.99, 1.15)	1.05 (0.98, 1.12)	0.084	0.19	0.059	**0.050**
	NDI	0.04 (0.006, 0.25)	0.048 (0.007, 0.31)	**6.73E-04**	**0.0015**	0.05 (0.009, 0.28)	0.08 (0.02, 0.35)	**5.75E-04**	**0.00103**	0.825	0.714
**Comorbidity Score**		1.66 (1.53, 1.79)	1.83 (1.71, 1.95)	**1.35E-37**	**4.25E-69**	1.61 (1.42, 1.82)	1.95 (1.76, 2.16)	**2.78E-14**	**1.80E-37**	0.664	0.281
**Comorbidities**	Respiratory	4.54 (3.49, 5.91)	5.08 (3.95, 6.55)	**1.50E-29**	**2.70E-36**	4.57 (3.08, 6.77)	5.05 (3.60, 7.10)	**4.18E-14**	**8.54E-21**	0.983	0.978
	Circulatory	2.55 (2.00, 3.26)	3.50 (2.78, 4.40)	**7.48E-14**	**6.84E-27**	3.40 (2.29, 5.03)	5.83 (4.15, 8.18)	**1.05E-09**	**2.09E-24**	0.217	**0.014**
	Any Cancer	1.08 (0.843, 1.4)	1.77 (1.41, 2.21)	0.528	**6.70E-07**	1.82 (1.19, 2.78)	2.96 (2.13, 4.12)	**5.36E-03**	**1.12E-10**	**3.51E-02**	**0.011**
	Type 2 Diabetes	2.05 (1.54, 2.71)	3.07 (2.38, 3.94)	**6.51E-07**	**3.07E-18**	1.63 (1.10, 2.40)	3.18 (2.32, 4.35)	**1.39E-02**	**4.82E-13**	0.342	0.855
	Kidney	3.22 (2.33, 4.46)	4.46 (3.36, 5.90)	**1.66E-12**	**1.99E-25**	2.16 (1.37, 3.40)	3.70 (2.59, 5.28)	**9.39E-04**	**5.51E-13**	0.152	0.422
	Liver	3.39 (2.32, 4.96)	4.77 (3.40, 6.70)	**2.89E-10**	**1.61E-19**	3.50 (1.56, 7.88)	6.16 (3.26, 11.6)	**2.44E-03**	**2.18E-08**	0.945	0.488

	Autoimmune	3.15 (2.38, 4.17)	3.67 (2.85, 4.71)	**8.83E-16**	**3.96E-24**	1.56 (1.02, 2.38)	3.49 (2.44, 5.00)	**4.16E-02**	**8.87E-12**	**6.38E-03**	0.825
**Hospitalized (1) vs not Hospitalized (0)**		**White Patients**				**Black Patients**				**White vs Black Patients**	
		**OR**		**P-value**		**OR**		**P-value**		**P-value**	
**Variables**		**Interaction**	**Stratified**	**Interaction**	**Stratified**	**Interaction**	**Stratified**	**Interaction**	**Stratified**	**Interaction**	**Stratified**
**Age (unit: 10-year)**		1.79 (1.52, 2.11)	1.73 (1.47, 2.05)	**2.54E-12**	**6.75E-11**	1.52 (1.27, 1.82)	1.59 (1.32, 1.92)	**3.46E-06**	**1.21E-06**	0.171	0.503
**Age Range** REF: [18,35)	[0,18)	0 (0, 0)	0 (0, 0)	0	0	1.71 (0.93, 5.99)	6.75 (0.49, 93.5)	0.632	0.154	0	0.000
	[35,50)	0.84 (0.30, 2.34)	0.33 (0.08, 1.27)	0.74	0.107	2.36 (0.93, 5.99)	1.04 (0.29, 3.71)	0.0717	0.947	0.144	0.220
	[50,65)	3.27 (1.42, 7.51)	0.51 (0.08, 3.37)	**0.00526**	0.485	3.54 (1.45, 8.62)	0.70 (0.095, 5.15)	**0.00546**	0.726	0.897	0.822
	[65,80)	7.22 (3.00, 17.4)	0.47 (0.03, 6.60)	**1.02E-05**	0.579	3.96 (1.50, 10.5)	0.31 (0.02, 5.51)	**0.00559**	0.425	0.359	0.831
	[80,100)	17.0 (5.64, 51)	0.44 (0.012, 16.1)	**4.63E-07**	0.655	114 (5.40, 2420)	4.32 (0.04, 464)	**0.00234**	0.54	0.248	0.448
**Male Sex**		1.4 (0.87, 2.27)	1.45 (0.89, 2.38)	0.169	0.137	2.71 (1.55, 4.75)	2.82 (1.64, 4.87)	**0.000487**	**0.000186**	0.0805	0.077
**BMI**		1.04 (1.01, 1.08)	1.04 (1.00, 1.07)	**0.0132**	**0.0425**	1.02 (0.99, 1.06)	1.02 (0.99, 1.06)	0.218	0.234	0.463	0.612
**BMI Range** REF: [18.5,25)	<18.5	1.05 (0.07, 17.1)	1.15 (0.07, 17.9)	0.971	0.922	12.8 (1.10, 22.8)	9.13 (0.24, 346)	0.169	0.233	0.285	0.372
	[25,30)	1.87 (0.90, 3.90)	2.10 (0.99, 4.46)	0.0951	**0.0548**	5.00 (1.10, 22.8)	4.67 (1.05, 20.8)	**0.0376**	**0.0427**	0.253	0.347
	>=30	2.39 (1.17, 4.89)	2.33 (1.11, 4.88)	**0.0169**	**0.0249**	4.44 (1.05, 18.7)	4.29 (1.04, 17.7)	**0.0421**	**0.0443**	0.448	0.455
**Ever-Smoker**		1.18 (0.71, 1.96)	1.15 (0.69, 1.93)	0.524	0.594	1.00 (0.56, 1.80)	1.06 (0.59, 1.9)	0.995	0.841	0.678	0.840
**Smoking status** REF: Never-Smoker	Past-Smoker	1.25 (0.75, 2.11)	1.22 (0.72, 2.07)	0.39	0.465	1.12 (0.61, 2.07)	1.25 (0.68, 2.32)	0.71	0.471	0.785	0.943
	Current-Smoker	0.69 (0.15, 3.27)	0.67 (0.14, 3.29)	0.642	0.623	0.51 (0.11, 2.36)	0.40 (0.09, 1.85)	0.389	0.242	0.786	0.648
**Alcohol Consumption**		1.18 (0.66, 2.11)	1.31 (0.71, 2.44)	0.576	0.388	0.66 (0.36, 1.22)	0.60 (0.33, 1.1)	0.184	0.0973	0.174	0.076
**SES**	Population density (1000-people/mi^2)	1.05 (0.93, 1.17)	1.07 (0.95, 1.21)	0.43	0.253	1.12 (0.98, 1.27)	1.11 (0.97, 1.27)	0.108	0.139	0.464	0.735
	NDI	0.52 (0.01, 19.9)	0.37 (0.009, 15.2)	0.723	0.6	7.72 (0.56, 106)	5.88 (0.444, 78)	0.126	0.179	0.239	0.231
**Comorbidity Score**		1.30 (1.11, 1.53)	1.30 (1.10, 1.53)	**0.00139**	**0.00184**	0.99 (0.83, 1.17)	1.00 (0.84, 1.20)	0.876	0.998	**0.0193**	**0.036**
**Comorbidities**	Respiratory	0.88 (0.47, 1.63)	0.85 (0.45, 1.59)	0.678	0.614	0.51 (0.25, 1.05)	0.55 (0.27, 1.12)	6.96E-02	0.0973	0.269	0.361
	Circulatory	1.85 (0.99, 3.46)	1.81 (0.96, 3.40)	5.34E-02	0.0663	0.85 (0.40, 1.79)	0.85 (0.40, 1.82)	0.662	0.677	0.114	0.136
	Any Cancer	1.09 (0.65, 1.84)	1.02 (0.60, 1.74)	0.744	0.929	0.72 (0.39, 1.31)	0.79 (0.43, 1.46)	0.276	0.452	0.291	0.530

	Type 2 Diabetes	2.59 (1.49, 4.48)	2.61 (1.50, 4.55)	**7.14E-04**	**0.0007**	1.17 (0.66, 2.06)	1.23 (0.69, 2.21)	0.593	0.487	**4.61E-02**	0.069
	Kidney	2.93 (1.61, 5.35)	2.92 (1.58, 5.39)	**4.57E-04**	**0.0006**	3.18 (1.63, 6.18)	3.17 (1.61, 6.24)	6.71E-04	0.000832	0.861	0.859
	Liver	1.70 (0.85, 3.38)	1.74 (0.88, 3.45)	0.131	0.111	0.46 (0.18, 1.14)	0.47 (0.19, 1.19)	9.24E-02	0.111	**2.44E-02**	**0.026**
	Autoimmune	1.76 (1.00, 3.07)	1.71 (0.97, 3.01)	**4.85E-02**	0.0615	0.84 (0.45, 1.55)	0.93 (0.50, 1.72)	0.574	0.82	**8.09E-02**	0.152

**ICU (1) vs not ICU (0)**		**White Patients**				**Black Patients**				**White vs Black Patients**	
		**OR**		**P-value**		**OR**		**P-value**		**P-value**	
**Variables**		**Interaction**	**Stratified**	**Interaction**	**Stratified**	**Interaction**	**Stratified**	**Interaction**	**Stratified**	**Interaction**	**Stratified**
**Age (unit: 10-year)**		1.55 (1.3, 1.86)	1.47 (1.23, 1.77)	**1.18E-06**	**3.54E-05**	1.32 (1.08, 1.61)	1.39 (1.12, 1.71)	**0.00614**	**0.00234**	0.221	0.6758
**Age Range** REF: [18,35)	[0,18)	0 (0, 0)	0 (0, 0)	0	0	4.17 (0.42, 5.1)	11.2 (0.62, 200)	0.223	0.101	0	0
	[35,50)	1.06 (0.288, 3.89)	0.41 (0.08, 2.14)	0.93	0.292	1.47 (0.42, 5.1)	0.92 (0.19, 4.46)	0.546	0.920	0.722	0.4896
	[50,65)	3.09 (1.05, 9.1)	0.48 (0.05, 4.35)	**0.041**	0.516	3.14 (1.02, 9.68)	1.17 (0.11, 12.4)	**0.0467**	0.898	0.984	0.5915
	[65,80)	5.21 (1.72, 15.8)	0.34 (0.02, 6.91)	**0.00351**	0.479	2.95 (0.89, 9.81)	0.60 (0.02, 16.6)	0.0781	0.760	0.486	0.8023
	[80,100)	8.6 (2.51, 29.4)	0.24 (0.004, 13.6)	**6.03E-04**	0.489	7.43 (1.60, 34.6)	1.02 (0.01, 77.9)	**0.0106**	0.994	0.883	0.6334
**Male Sex**		1.47 (0.85, 2.56)	1.54 (0.87, 2.72)	0.169	0.137	3.20 (1.69, 6.07)	3.41 (1.8, 6.45)	**3.62E-04**	**1.63E-04**	0.072	0.0686
**BMI**		1.03 (0.99, 1.07)	1.02 (0.98, 1.06)	0.0798	0.284	1.03 (0.99, 1.08)	1.03 (0.99, 1.08)	0.151	0.159	0.95	0.7070
**BMI Range** REF: [18.5,25)	<18.5	4.57 (0.31, 67.8)	5.35 (0.39, 74.3)	0.269	0.212	0 (0.45, 11.8)	0 (0, 0)	0	0	0	0
	[25,30)	1.74 (0.72, 4.21)	2.07 (0.84, 5.12)	0.223	0.117	2.29 (0.45, 11.8)	2.29 (0.45, 11.7)	0.32	0.317	0.769	0.9124
	>=30	2.56 (1.08, 6.05)	2.41 (0.99, 5.84)	**0.033**	**0.0512**	1.95 (0.41, 9.33)	1.95 (0.41, 9.28)	0.405	0.403	0.765	0.8155
**Ever-Smoker**		1.33 (0.75, 2.36)	1.26 (0.70, 2.27)	0.334	0.441	1.16 (0.60, 2.23)	1.27 (0.65, 2.47)	0.656	0.486	0.760	0.9904
**Smoking status** REF: Never-Smoker	Past-Smoker	1.41 (0.79, 2.52)	1.34 (0.73, 2.43)	0.249	0.344	1.26 (0.64, 2.47)	1.45 (0.72, 2.92)	0.505	0.294	0.799	0.8577
	Current-Smoker	0.82 (0.13, 5.35)	0.73 (0.103, 5.12)	0.833	0.749	0.75 (0.12, 4.74)	0.58 (0.09, 3.7)	0.756	0.562	0.946	0.8660
**Alcohol Consumption**		1.03 (0.54, 1.97)	1.22 (0.616, 2.4)	0.919	0.571	0.98 (0.49, 1.97)	0.89 (0.43, 1.76)	0.955	0.693	0.911	0.4980
**SES**	Population density (1000-people/mi^2)	1.05 (0.92, 1.19)	1.08 (0.94, 1.24)	0.485	0.293	1.09 (0.95, 1.26)	1.07 (0.92, 1.24)	0.227	0.361	0.647	0.9579
	NDI	1.23 (0.02, 72)	0.73 (0.01, 47)	0.921	0.882	34.4 (1.9, 623)	32.1 (1.71, 603)	**0.0166**	**0.021**	0.191	0.1456
**Comorbidity Score**		1.43 (1.19, 1.73)	1.42 (1.18, 1.71)	**1.74E-04**	**2.74E-04**	1.00 (0.83, 1.21)	1.00 (0.82, 1.23)	0.986	0.971	**0.008**	**0.0145**

**Comorbidities**	Respiratory	2.23 (0.96, 5.19)	2.20 (0.94, 5.13)	**6.25E-02**	**0.0686**	0.51 (0.24, 1.09)	0.54 (0.25, 1.15)	8.21E-02	0.109	**1.09E-02**	**0.015**
	Circulatory	2.08 (0.94, 4.61)	2.01 (0.91, 4.47)	7.03E-02	0.0854	0.92 (0.37, 2.28)	0.90 (0.36, 2.27)	0.858	0.823	0.181	0.196
	Any Cancer	1.47 (0.82, 2.63)	1.34 (0.75, 2.41)	0.195	0.328	0.53 (0.26, 1.06)	0.54 (0.26, 1.12)	7.37E-02	0.0998	2.50E-02	0.058
	Type 2 Diabetes	2.21 (1.22, 4.00)	2.16 (1.19, 3.92)	**8.98E-03**	**0.0118**	1.30 (0.69, 2.43)	1.34 (0.70, 2.58)	0.417	0.376	0.221	0.294
	Kidney	3.16 (1.70, 5.87)	3.01 (1.60, 5.63)	**2.71E-04**	**0.000596**	2.84 (1.47, 5.49)	2.81 (1.41, 5.64)	**1.95E-03**	**0.0035**	0.814	0.890
	Liver	1.47 (0.68, 3.18)	1.54 (0.72, 3.3)	0.327	0.264	0.42 (0.13, 1.29)	0.40 (0.13, 1.28)	0.129	0.123	7.11E-02	0.057
	Autoimmune	2.10 (1.13, 3.91)	2.05 (1.10, 3.81)	**1.86E-02**	**0.0239**	1.16 (0.59, 2.30)	1.29 (0.64, 2.58)	0.669	0.48	0.204	0.329

**Deceased (1) vs Alive (0)**		**White Patients**				**Black Patients**				**White vs Black Patients**	
		**OR**		**P-value**		**OR**		**P-value**		**P-value**	
**Variables**		**Interaction**	**Stratified**	**Interaction**	**Stratified**	**Interaction**	**Stratified**	**Interaction**	**Stratified**	**Interaction**	**Stratified**
**Age (unit: 10-year)**		2.7 (1.84, 3.96)	2.64 (1.81, 3.86)	**3.94E-07**	**5.18E-07**	1.74 (1.19, 2.54)	1.80 (1.22, 2.67)	**0.0041**	**0.00314**	0.109	0.170
**Age Range** REF: [18,35)	[0,18)	0 (0, 0)	0 (0, 0)	0	0	0 (0.16, 75.3)	29.9 (0.16, 5640)	0	0.204		
	[35,50)	0.27 (0.01, 6.48)	0.24 (0.007, 8.29)	0.415	0.428	3.47 (0.16, 75.3)	3.19 (0.11, 90.6)	0.428	0.496	0.255	0.297
	[50,65)	0.44 (0.05, 4.33)	0.36 (0.007, 19.7)	0.483	0.619	4.60 (0.25, 86.2)	2.75 (0.04, 210)	0.307	0.648	0.213	0.501
	[65,80)	3.21 (0.52, 19.7)	2.37 (0.0138, 409)	0.207	0.742	7.33 (0.38, 140)	3.22 (0.01, 886)	0.186	0.683	0.637	0.937
	[80,100)	22.1 (3.64, 134)	14.3 (0.015, 13400)	**0.000771**	0.446	31.9 (1.45, 701)	10.9 (0.01, 11800)	**0.0281**	0.504	0.84	0.956
**Male Sex**		2.52 (0.97, 6.53)	2.57 (0.977, 6.74)	0.057	0.0559	4.17 (1.32, 13.2)	3.92 (1.29, 11.9)	**0.0151**	**0.0159**	0.509	0.572
**BMI**		1.04 (0.98, 1.1)	1.04 (0.977, 1.11)	0.195	0.219	1.08 (1.00, 1.16)	1.07 (0.99, 1.15)	**0.0363**	0.095	0.413	0.615
**BMI Range** REF: [18.5,25)	<18.5	11.6 (0.36, 369)	10.1 (0.295, 344)	0.165	0.199	0 (0.10, 82.8)	0 (0, 0)	0	0		
	[25,30)	0.93 (0.22, 3.92)	1.13 (0.255, 4.99)	0.926	0.874	2.89 (0.10, 82.8)	2.95 (0.11, 77.8)	0.536	0.517	0.545	0.600
	>=30	2.26 (0.56, 9.19)	2.63 (0.596, 11.6)	0.254	0.201	7.12 (0.28, 184)	5.85 (0.24, 142)	0.237	0.278	0.525	0.657
**Ever-Smoker**		1.13 (0.43, 2.96)	1.01 (0.37, 2.74)	0.809	0.99	1.22 (0.41, 3.59)	1.61 (0.54, 4.77)	0.723	0.392	0.916	0.534
**Smoking status** REF: Never-Smoker	Past-Smoker	1.19 (0.45, 3.13)	1.06 (0.389, 2.89)	0.726	0.909	1.27 (0.43, 3.77)	1.75 (0.58, 5.29)	0.667	0.323	0.928	0.512
	Current-Smoker	1.21 (0.04, 37.2)	0.91 (0.02, 41.6)	0.915	0.961	2.8 (0.12, 64.3)	1.7 (0.08, 36.2)	0.519	0.735	0.721	0.803
**Alcohol Consumption**		1.24 (0.42, 3.69)	1.32 (0.42, 4.2)	0.693	0.633	1.34 (0.44, 4.13)	1.29 (0.43, 3.83)	0.607	0.65	0.923	0.972
**SES**	Population density (1000-people/mi^2)	1.04 (0.85, 1.27)	1.07 (0.85, 1.34)	0.727	0.555	1.18 (0.94, 1.49)	1.14 (0.91, 1.43)	0.147	0.252	0.373	0.698

	NDI	0.48 (0.0004, 526)	0.35 (0.0003, 480)	0.837	0.777	228 (2, 26000)	130 (1.25, 13500)	**0.0246**	**0.0399**	0.153	0.177
**Comorbidity Score**		1.37 (1.01, 1.84)	1.37 (1.02, 1.85)	**0.0409**	**0.0395**	1.21 (0.87, 1.69)	1.25 (0.91, 1.74)	0.246	0.173	0.603	0.696
**Comorbidities**	Respiratory	1.38 (0.38, 4.96)	1.52 (0.41, 5.61)	0.623	0.526	0.71 (0.21, 2.40)	0.77 (0.23, 2.55)	0.576	0.669	0.458	0.450
	Circulatory	0.90 (0.25, 3.18)	0.88 (0.24, 3.2)	0.868	0.842	1.18 (0.18, 7.79)	1.09 (0.18, 6.57)	0.864	0.921	0.814	0.844
	Any Cancer	2.23 (0.88, 5.63)	2.02 (0.80, 5.13)	9.03E-02	0.139	1.48 (0.54, 4.10)	1.73 (0.63, 4.79)	0.446	0.29	0.56	0.827
	Type 2 Diabetes	3.07 (1.22, 7.75)	3.22 (1.25, 8.27)	**1.73E-02**	**0.0151**	1.89 (0.67, 5.37)	2.00 (0.72, 5.62)	0.23	0.186	0.494	0.506
	Kidney	3.68 (1.44, 9.41)	3.52 (1.37, 9.03)	**6.59E-03**	**0.00902**	3.31 (1.11, 9.85)	3.35 (1.12, 9.97)	**3.14E-02**	**0.0299**	0.886	0.947
	Liver	1.49 (0.42, 5.33)	1.87 (0.52, 6.78)	0.539	0.341	0.69 (0.15, 3.25)	0.73 (0.16, 3.33)	0.64	0.685	0.454	0.355
	Autoimmune	0.58 (0.16, 2.02)	0.61 (0.18, 2.15)	0.389	0.445	1.12 (0.37, 3.44)	1.22 (0.41, 3.68)	0.838	0.718	0.434	0.417

Abbreviations: OR, odds ratio; ICU, intensive care unit; BMI, body mass index; NA, not applicable; REF, reference group; SES, social economics status; NDI, 2010 Neighborhood Socioeconomic Disadvantage Index.

* the race/ethnicity-stratified used frequency-matched, instead of unmatched comparison group, as the proportion of race/ethnicity in comparison group are not comparable to the stratified study population.

eTable 4. Sensitivity Analysis Using Patients With Primary Care at MM

		Susceptibility		Prognosis			
		Positive (1) vs. Comparison Group (0)		Hospitalized (1) vs. Not (0)		ICU (1) vs. Not (0)	
		Full Cohort	Primary Care at MM	Full Cohort	Primary Care at MM	Full Cohort	Primary Care at MM
		(n_0=5611, n_1=761)	(n_0=987, n_1=510)	(n_0=486, n_1=270)	(n_0=370, n_1=138)	(n_0=615, n_1=141)	(n_0=435, n_1=73)
Variable		OR (95% CI)					
**Age (unit: 10-year)**		1.09 (1.05, 1.14)	1.29 (1.19, 1.39)	1.72 (1.53, 1.93)	1.68 (1.43, 1.97)	1.45 (1.27, 1.65)	1.29 (1.08, 1.54)
**Age Range** REF: [18, 35)	[0,18)	0.06 (0.03, 0.15)	0.09 (0.03, 0.27)	5.01 (0.57, 44.3)	15.0 (1.27, 179)	8.75 (0.90, 85.2)	15.6 (1.19, 203)
	[35,50)	1.56 (1.03, 2.37)	1.01 (0.56, 1.83)	0.75 (0.32, 1.77)	0.39 (0.13, 1.22)	0.72 (0.25, 2.1)	0.68 (0.17, 2.7)
	[50,65)	1.59 (0.82, 3.11)	0.77 (0.30, 2.01)	0.72 (0.20, 2.53)	0.19 (0.03, 1.03)	0.86 (0.19, 3.87)	0.70 (0.09, 5.18)
	[65,80)	1.20 (0.46, 3.09)	0.33 (0.08, 1.31)	0.58 (0.097, 3.44)	0.16 (0.01, 1.78)	0.71 (0.09, 5.68)	0.69 (0.04, 11.5)
	[80,100)	1.32 (0.38, 4.60)	0.26 (0.04, 1.70)	1.15 (0.10, 13.1)	0.21 (0.008, 5.94)	0.64 (0.04, 10.1)	0.79 (0.02, 34.9)
**Male Sex**		0.90 (0.76, 1.07)	0.87 (0.67, 1.14)	1.91 (1.36, 2.68)	1.98 (1.26, 3.13)	2.25 (1.52, 3.34)	2.62 (1.54, 4.48)
**BMI**		1.03 (1.02, 1.04)	1.02 (1.00, 1.04)	1.04 (1.01, 1.06)	1.04 (1.01, 1.07)	1.03 (1.01, 1.06)	1.03 (0.99, 1.07)
**BMI Range** REF: [18.5, 25)	<18.5	0.65 (0.27, 1.54)	0.68 (0.18, 2.64)	1.8 (0.24, 13.6)	0 (0, 0)	2.09 (0.22, 20.3)	0 (0, 0)
	[25, 30)	1.62 (1.25, 2.10)	1.25 (0.87, 1.80)	2.20 (1.23, 3.94)	1.79 (0.82, 3.91)	1.59 (0.79, 3.21)	0.66 (0.28, 1.58)
	>=30	1.70 (1.33, 2.18)	1.45 (1.02, 2.06)	2.43 (1.38, 4.30)	2.36 (1.11, 5.05)	2.17 (1.10, 4.26)	1.14 (0.51, 2.54)
**Ever-Smoker**		0.73 (0.59, 0.89)	1.02 (0.76, 1.38)	1.11 (0.77, 1.60)	1.41 (0.882, 2.26)	1.36 (0.90, 2.07)	1.82 (1.06, 3.12)
**Smoking Status** REF: Never	Past	0.92 (0.74, 1.14)	1.10 (0.80, 1.52)	1.21 (0.83, 1.77)	1.52 (0.934, 2.47)	1.47 (0.96, 2.24)	1.92 (1.11, 3.33)
	Current	0.31 (0.20, 0.48)	0.70 (0.37, 1.32)	0.51 (0.17, 1.52)	0.81 (0.223, 2.93)	0.67 (0.17, 2.64)	1.23 (0.28, 5.4)
**Alcohol Consumption**		1.58 (1.29, 1.95)	1.11 (0.83, 1.49)	0.83 (0.56, 1.23)	0.67 (0.405, 1.09)	1.1 (0.69, 1.74)	1.31 (0.72, 2.37)
**Race/Ethnicity** REF: White	Black	6.11 (4.83, 7.73)	4.09 (2.81, 5.94)	1.72 (1.15, 2.58)	2.08 (1.21, 3.56)	1.15 (0.73, 1.82)	0.89 (0.47, 1.66)
	Other	1.67 (1.26, 2.23)	1.29 (0.88, 1.89)	1.42 (0.79, 2.54)	2.43 (1.2, 4.91)	0.86 (0.42, 1.78)	1.01 (0.43, 2.4)
	Unknown	0.11 (0.076, 0.17)	0.04 (0.02, 0.07)	0.72 (0.28, 1.84)	0.78 (0.10, 6.1)	0.60 (0.18, 2.04)	0.46 (0.02, 10.2)

**SES**	NDI	0.04 (0.012, 0.14)	1.01 (0.95, 1.07)	5.51 (0.74, 41.1)	1.06 (0.94, 1.19)	13.7 (1.46, 128)	1.11 (0.96, 1.27)
	Population density (1000-people/mi^2)	1.12 (1.08, 1.16)	4.51 (0.64, 32.1)	1.10 (1.01, 1.19)	2.64 (0.17, 40.5)	1.08 (0.99, 1.19)	3.30 (0.14, 79.1)
**Comorbidity Score**		1.64 (1.54, 1.75)	1.14 (1.03, 1.27)	1.15 (1.03, 1.29)	1.36 (1.16, 1.60)	1.16 (1.02, 1.32)	1.44 (1.19, 1.74)
**Comorbidities**	Respiratory	4.09 (3.36, 4.97)	0.96 (0.67, 1.36)	0.81 (0.53, 1.23)	1.16 (0.59, 2.27)	0.98 (0.60, 1.61)	1.35 (0.58, 3.11)
	Circulatory	2.85 (2.34, 3.47)	1.32 (0.96, 1.82)	1.35 (0.87, 2.08)	2.09 (1.03, 4.25)	1.21 (0.71, 2.05)	2.16 (0.90, 5.21)
	Any Cancer	1.18 (0.96, 1.45)	1.05 (0.76, 1.45)	0.90 (0.61, 1.32)	1.30 (0.80, 2.13)	0.93 (0.60, 1.44)	1.34 (0.76, 2.36)
	Type 2 Diabetes	2.01 (1.61, 2.50)	1.33 (0.91, 1.93)	1.82 (1.25, 2.64)	1.98 (1.21, 3.25)	1.50 (0.99, 2.28)	2.43 (1.37, 4.33)
	Kidney	2.82 (2.18, 3.66)	1.32 (0.86, 2.04)	2.87 (1.87, 4.42)	3.68 (2.13, 6.34)	2.74 (1.76, 4.26)	4.00 (2.18, 7.32)
	Liver	3.33 (2.42, 4.57)	1.68 (1.05, 2.70)	0.997 (0.59, 1.68)	1.02 (0.53, 1.95)	0.95 (0.52, 1.73)	0.70 (0.31, 1.58)
	Autoimmune	2.44 (1.94, 3.06)	1.42 (1.01, 1.98)	1.24 (0.83, 1.85)	1.68 (1.02, 2.78)	1.45 (0.92, 2.29)	2.14 (1.21, 3.79)

Abbreviations: OR, odds ratio; ICU, intensive care unit; BMI, body mass index; NA, not applicable; REF, reference group; SES, social economics status; NDI, 2010 Neighborhood Socioeconomic Disadvantage Index.

eTable 5. Observed Missingness Across Variables and Descriptive Characteristics of the COVID-19 Tested or Diagnosed Cohort of the Full Cohort and White and Black Patients

eTable 5A. Observed Missingness across Variables of the Full Cohort, White and Black Patients

Full Cohort	**COVID-19 Tested**						**Comparison Group**
	**Overall**	**Negative**	**COVID-19 Positive**				
			**Overall**	**Hospitalized**	**ICU**	**Deceased**	
	**(n=5,698)**	**(n=4,559)**	**(n=1,139)**	**(n=523)**	**(n=283)**	**(n=88)**	**(n=7,168)**
**Variable**	**n (%)**						
**Age/Age Range**	0 (0.0)	0 (0.0)	0 (0.0)	0 (0.0)	0 (0.0)	0 (0.0)	3 (0.0)
**Male Sex**	1 (0.0)	1 (0.0)	0 (0.0)	0 (0.0)	0 (0.0)	0 (0.0)	6 (0.1)
**Primary Care in MM**	0 (0.0)	0 (0.0)	0 (0.0)	0 (0.0)	0 (0.0)	0 (0.0)	0 (0.0)
**BMI/BMI Range**	762 (13.4)	672 (14.7)	90 (7.9)	24 (4.6)	9 (3.2)	4 (4.5)	3,171 (44.2)
**Ever-Smoker/ Smoking Status**	605 (10.6)	426 (9.3)	179 (15.7)	97 (18.5)	79 (27.9)	35 (39.8)	1,839 (25.7)
**Alcohol Consumption**	1,538 (27.0)	1,123 (24.6)	415 (36.4)	251 (48.0)	146 (51.6)	47 (53.4)	3,331 (46.5%)
**Race Ethnicity***	356 (6.2)	272 (6)	84 (7.4)	49 (9.4)	31 (11)	14 (15.9)	2673 (37.3)
**SES (NDI/Population density)**	1,105 (19.4)	770 (16.9)	335 (29.4)	240 (45.9)	136 (48.1)	40 (45.5)	1,257 (17.5%)
**Comorbidity Score**	700 (12.3)	440 (9.7)	260 (22.8)	176 (33.7)	98 (34.6)	23 (26.1)	400 (5.6%)
White	**(n=3,740)**	**(n=3,248)**	**(n=492)**	**(n=190)**	**(n=101)**	**(n=35)**	**(n=10,126)**
**Age/Age Range**	0 (0.0)	0 (0.0)	0 (0.0)	0 (0.0)	0 (0.0)	0 (0.0)	0 (0.0)
**Male Sex**	0 (0.0)	0 (0.0)	0 (0.0)	0 (0.0)	0 (0.0)	0 (0.0)	0 (0.0)
**Primary Care in MM**	0 (0.0)	0 (0.0)	0 (0.0)	0 (0.0)	0 (0.0)	0 (0.0)	0 (0.0)
**BMI/BMI Range**	436 (11.7)	410 (12.6)	26 (5.3)	7 (3.7)	2 (2.0)	1 (2.9)	3951 (39.0)
**Ever-Smoker/ Smoking Status**	260 (7.0)	217 (6.7)	43 (8.7)	20 (10.5)	13 (12.9)	7 (20.0)	2086 (20.6)
**Alcohol Consumption**	820 (21.9)	695 (21.4)	125 (25.4)	66 (34.7)	31 (30.7)	12 (34.3)	4104 (40.5)
**SES (NDI/Population density)**	626 (16.7)	534 (16.4)	92 (18.7)	63 (33.2)	32 (31.7)	12 (34.3)	1668 (16.5)
**Comorbidity Score**	334 (8.9)	269 (8.3)	65 (13.2)	45 (23.7)	23 (22.8)	7 (20.0)	658 (6.5)
Black	**(n=1,058)**	**(n=616)**	**(n=442)**	**(n=233)**	**(n=132)**	**(n=36)**	**(n=1,178)**
**Age/Age Range**	0 (0.0)	0 (0.0)	0 (0.0)	0 (0.0)	0 (0.0)	0 (0.0)	0 (0.0)
**Male Sex**	0 (0.0)	0 (0.0)	0 (0.0)	0 (0.0)	0 (0.0)	0 (0.0)	0 (0.0)
**Primary Care in MM**	0 (0.0)	0 (0.0)	0 (0.0)	0 (0.0)	0 (0.0)	0 (0.0)	0 (0.0)
**BMI/BMI Range**	121 (11.4)	84 (13.6)	37 (8.4)	9 (3.9)	6 (4.5)	0 (0)	531 (45.1)
**Ever-Smoker/ Smoking Status**	127 (12.0)	52 (8.4)	75 (17.0)	43 (18.5)	39 (29.5)	14 (38.9)	280 (23.8)
**Alcohol Consumption**	325 (30.7)	142 (23.1)	183 (41.4)	121 (51.9)	78 (59.1)	20 (55.6)	519 (44.1)
**SES (NDI/Population density)**	244 (23.1)	89 (14.4)	155 (35.1)	115 (49.4)	69 (52.3)	17 (47.2)	158 (13.4)
**Comorbidity Score**	184 (17.4)	57 (9.3)	127 (28.7)	87 (37.3)	53 (40.2)	12 (33.3)	122 (10.4)

* In the main analyses unknown race and/or ethnicity were combined into the category "Unknown race/ethnicity" to retain sample size

eTable 5B. Descriptive Characteristics of the COVID-19 Tested or Diagnosed Cohort Stratified by White and Black Patients

**White Patients**		**Total Tested for COVID-19**						**Matched Comparison Group (n=10,126)**
		**Overall (n=1,058)**	**Negative (n=616)**	**Tested Positive**				
				**Overall (n=442)**	**Hospitalized (n=233)**	**ICU (n=132)**	**Deceased (n=36)**	
**Variable**		**n (%)**						
**Age (in years)**	mean (SD)	47.1 (21.0)	46.1 (21.2)	53.6 (18.4)	64.5 (15.8)	63.8 (15.8)	76.0 (14.3)	44.8 (24.2)
	median [IQR]	47.0 [31.0]	46.0 [31.0]	54.0 [28.0]	65.0 [21.0]	65.0 [18.0]	80.0 [15.0]	45.0 [41.0]
**Age Range**	[0,18)	258 (6.9)	257 (7.9)	1 (0.2)	0 (0)	0 (0)	0 (0)	1560 (15.4)
	[18,35)	873 (23.3)	777 (23.9)	96 (19.5)	13 (6.8)	9 (8.9)	2 (5.7)	2358 (23.3)
	[35,50)	864 (23.1)	766 (23.6)	98 (19.9)	14 (7.4)	6 (5.9)	0 (0)	1568 (15.5)
	[50,65)	914 (24.4)	765 (23.6)	149 (30.3)	64 (33.7)	34 (33.7)	2 (5.7)	2075 (20.5)
	[65,80)	606 (16.2)	504 (15.5)	102 (20.7)	63 (33.2)	35 (34.7)	11 (31.4)	1790 (17.7)
	[80,100)	225 (6.0)	179 (5.5)	46 (9.3)	36 (18.9)	17 (16.8)	20 (57.1)	775 (7.7)
**Male Sex**		1427 (38.2)	1182 (36.4)	245 (49.8)	114 (60.0)	62 (61.4)	25 (71.4)	3889 (38.4)
**Primary Care in MM**		2221 (59.4)	1929 (59.4)	292 (59.3)	73 (38.4)	48 (47.5)	14 (40.0)	985 (9.7)
**BMI, mean (SD)**		29.4 (7.42)	29.2 (7.37)	30.5 (7.69)	30.9 (8.28)	30.7 (8.51)	30.1 (7.94)	28.6 (8.14)
**BMI Range**	<18.5	56 (1.5)	52 (1.6)	4 (0.8)	2 (1.1)	2 (2.0)	1 (2.9)	136 (1.3)
	[18.5, 25)	950 (25.4)	848 (26.1)	102 (20.7)	32 (16.8)	18 (17.8)	6 (17.1)	1988 (19.6)
	[25, 30)	1009 (27.0)	849 (26.1)	160 (32.5)	69 (36.3)	36 (35.6)	13 (37.1)	1965 (19.4)
	>=30	1289 (34.5)	1089 (33.5)	200 (40.7)	80 (42.1)	43 (42.6)	14 (40.0)	2086 (20.6)
**Ever-Smoker**		1420 (38.0)	1254 (38.6)	166 (33.7)	77 (40.5)	43 (42.6)	16 (45.7)	2821 (27.9)
**Smoking Status**	Never	2060 (55.1)	1777 (54.7)	283 (57.5)	93 (48.9)	45 (44.6)	12 (34.3)	5219 (51.5)
	Past	1134 (30.3)	986 (30.4)	148 (30.1)	72 (37.9)	41 (40.6)	16 (45.7)	1923 (19.0)
	Current	286 (7.6)	268 (8.3)	18 (3.7)	5 (2.6)	2 (2.0)	0 (0)	898 (8.9)
**Alcohol Consumption**		855 (22.9)	1804 (55.5)	261 (53.0)	91 (47.9)	50 (49.5)	18 (51.4)	3392 (33.5)
**SES, mean (SD)**	NDI	0.0895 (0.0628)	0.0891 (0.0622)	0.0920 (0.0661)	0.0909 (0.0565)	0.0925 (0.0587)	0.0890 (0.0586)	0.0965 (0.0631)
	Population density (1000-people/mi^2)	2,340 (2,230)	2,290 (2,220)	2,680 (2,210)	2,960 (2,430)	3,010 (2,370)	3,190 (2,500)	2,020 (2,410)
**Comorbidity Score, mean (SD)**		2.57 (1.57)	2.56 (1.57)	2.61 (1.58)	3.30 (1.62)	3.58 (1.61)	3.96 (1.75)	1.31 (1.24)

Black patients		Total Tested for COVID-19						Matched Comparison Group (n=1,178)
		Overall (n=1,058)	Negative (n=616)	Tested Positive				
				Overall (n=442)	Hospitalized (n=233)	ICU (n=132)	Deceased (n=36)	
**Variable**		n (%)						
**Age (in years)**	mean (SD)	47.8 (19.6)	45.2 (20.5)	51.5 (17.5)	57.6 (16.8)	57.6 (16.8)	68.4 (10.7)	39.0 (22.6)
	median [IQR]	49.0 [29.0]	45.5 [31.0]	52.0 [26.0]	59.0 [23.0]	59.0 [21.3]	68.5 [18.5]	36.0 [38.0]
**Age Range**	[0,18)	59 (5.6%)	51 (8.3%)	8 (1.8%)	4 (1.7%)	3 (2.3%)	0 (0%)	244 (20.7%)
	[18,35)	214 (20.2%)	139 (22.6%)	75 (17.0%)	18 (7.7%)	13 (9.8%)	0 (0%)	314 (26.7%)
	[35,50)	273 (25.8%)	165 (26.8%)	108 (24.4%)	48 (20.6%)	18 (13.6%)	2 (5.6%)	211 (17.9%)
	[50,65)	283 (26.7%)	140 (22.7%)	143 (32.4%)	82 (35.2%)	52 (39.4%)	12 (33.3%)	219 (18.6%)
	[65,80)	180 (17.0%)	99 (16.1%)	81 (18.3%)	55 (23.6%)	34 (25.8%)	13 (36.1%)	145 (12.3%)
	[80,100)	49 (4.6%)	22 (3.6%)	27 (6.1%)	26 (11.2%)	12 (9.1%)	9 (25.0%)	45 (3.8%)
**Male Sex**		390 (36.9%)	195 (31.7%)	195 (44.1%)	128 (54.9%)	77 (58.3%)	23 (63.9%)	407 (34.6%)
**Primary Care in MM**		526 (49.7%)	327 (53.1%)	199 (45.0%)	63 (27.0%)	27 (20.5%)	8 (22.2%)	144 (12.2%)
**BMI, mean (SD)**		32.8 (9.45)	31.3 (8.22)	34.7 (10.6)	35.1 (12.5)	35.8 (14.6)	33.1 (6.89)	31.1 (14.7)
**BMI Range**	<18.5	15 (1.4%)	11 (1.8%)	4 (0.9%)	3 (1.3%)	0 (0%)	0 (0%)	16 (1.4%)
	[18.5, 25)	138 (13.0%)	106 (17.2%)	32 (7.2%)	14 (6.0%)	11 (8.3%)	3 (8.3%)	165 (14.0%)
	[25, 30)	245 (23.2%)	144 (23.4%)	101 (22.9%)	65 (27.9%)	34 (25.8%)	12 (33.3%)	171 (14.5%)
	>=30	539 (50.9%)	271 (44.0%)	268 (60.6%)	142 (60.9%)	81 (61.4%)	21 (58.3%)	295 (25.0%)
**Ever-Smoker**		340 (32.1%)	219 (35.6%)	121 (27.4%)	68 (29.2%)	37 (28.0%)	15 (41.7%)	276 (23.4%)
**Smoking Status**	Never	591 (55.9%)	345 (56.0%)	246 (55.7%)	122 (52.4%)	56 (42.4%)	7 (19.4%)	622 (52.8%)
	Past	248 (23.4%)	141 (22.9%)	107 (24.2%)	66 (28.3%)	36 (27.3%)	15 (41.7%)	166 (14.1%)
	Current	92 (8.7%)	78 (12.7%)	14 (3.2%)	2 (0.9%)	1 (0.8%)	0 (0%)	110 (9.3%)
**Alcohol Consumption**		428 (40.5%)	270 (43.8%)	158 (35.7%)	63 (27.0%)	32 (24.2%)	9 (25.0%)	283 (24.0%)
**SES, mean (SD)**	NDI	0.174 (0.0995)	0.173 (0.0974)	0.176 (0.104)	0.183 (0.109)	0.202 (0.106)	0.211 (0.0897)	0.197 (0.113)
	Population density (1000-people/mi^2)	3630 (2370)	3580 (2450)	3730 (2200)	4080 (2270)	4250 (2250)	4850 (2120)	3670 (2430)
**Comorbidity Score, mean (SD)**		2.86 (1.62)	2.83 (1.63)	2.91 (1.60)	3.26 (1.59)	3.32 (1.52)	4.17 (1.27)	1.37 (1.29)

Abbreviations: MM, Michigan Medicine; ICU, intensive care unit; BMI, body mass index; MM, Michigan Medicine; SES, social economics status; NDI, 2010 Neighborhood Socioeconomic Disadvantage Index.

© 2020 Gu T et al. *JAMA Network Open.*

eTable 6. Odds Ratio of Susceptibility From Logistic Regression for Full Cohort

**Tested (1) vs. Comparison Group (0)**		**Unadjusted (n_0=7165, n_1=5698)**	**Adjustment 1 (n_0=5910, n_1=4593)**	**Adjustment 2 (n_0=5909, n_1=4593)**	**Adjustment 3 (n_0=5611, n_1=4417)**
Variable		OR (95% CI)			
**Age (unit: 10-year)**		1.09 (1.07, 1.10)	1.13 (1.11, 1.16)	1.13 (1.11, 1.16)	0.97 (0.95, 0.99)
**Age Range** REF: [18,35)	[0,18)	0.38 (0.33, 0.44)	0.37 (0.29, 0.47)	0.38 (0.30, 0.48)	0.34 (0.26, 0.44)
	[35,50)	1.48 (1.33, 1.65)	1.38 (1.13, 1.67)	1.36 (1.12, 1.65)	1.31 (1.06, 1.62)
	[50,65)	1.25 (1.13, 1.39)	1.04 (0.75, 1.43)	1.03 (0.75, 1.42)	0.82 (0.58, 1.17)
	[65,80)	0.99 (0.88, 1.10)	0.82 (0.52, 1.29)	0.80 (0.51, 1.26)	0.60 (0.36, 0.98)
	[80,100)	1.01 (0.87, 1.19)	0.81 (0.44, 1.47)	0.78 (0.43, 1.42)	0.72 (0.37, 1.38)
**Male Sex**		0.74 (0.69, 0.79)	0.64 (0.59, 0.70)	0.64 (0.58, 0.70)	0.63 (0.57, 0.69)
**BMI**		1.03 (1.02, 1.03)	1.02 (1.01, 1.02)	1.02 (1.01, 1.03)	1.01 (0.99, 1.01)
**BMI Range** REF: [18.5,25)	<18.5	0.99 (0.73, 1.36)	0.84 (0.58, 1.20)	0.84 (0.59, 1.21)	0.72 (0.49, 1.06)
	[25,30)	1.24 (1.12, 1.39)	1.25 (1.1, 1.43)	1.27 (1.12, 1.45)	1.23 (1.07, 1.41)
	>=30	1.55 (1.4, 1.72)	1.35 (1.19, 1.53)	1.41 (1.25, 1.60)	1.14 (1.00, 1.31)
**Ever-Smoker**		1.37 (1.27, 1.49)	1.14 (1.03, 1.26)	1.20 (1.08, 1.32)	1.05 (0.94, 1.18)
**Smoking Status** REF: Never-Smoker	Past-Smoker	1.64 (1.5, 1.8)	1.40 (1.25, 1.57)	1.44 (1.28, 1.61)	1.22 (1.07, 1.38)
	Current-Smoker	0.87 (0.76, 0.99)	0.71 (0.60, 0.83)	0.76 (0.65, 0.90)	0.75 (0.63, 0.89)
**Alcohol Consumption**		1.54 (1.40, 1.68)	1.65 (1.49, 1.84)	1.60 (1.44, 1.79)	1.82 (1.62, 2.04)
**Race/Ethnicity** REF: White	Black	2.58 (2.28, 2.93)	2.16 (1.87, 2.48)	2.62 (2.26, 3.05)	2.31 (1.96, 2.73)
	Other / Known Ethnicity	0.98 (0.86, 1.11)	1.01 (0.88, 1.17)	1.01 (0.87, 1.17)	1.09 (0.93, 1.28)
	Other / Unknown Ethnicity	0.13 (0.11, 0.14)	0.09 (0.08, 0.11)	0.09 (0.08, 0.11)	0.11 (0.097, 0.13)
**SES**	Population density (1000-people/mi^2)	1.05 (1.04, 1.07)	1.04 (1.02, 1.06)	1.07 (1.05, 1.09)	1.08 (1.06, 1.11)
	NDI	0.81 (0.50, 1.34)	0.07 (0.04, 0.14)	0.07 (0.04, 0.14)	0.04 (0.02, 0.07)
**Comorbidity Score**		1.86 (1.81, 1.92)	1.81 (1.74, 1.88)	1.82 (1.75, 1.89)	1.82 (1.75, 1.89)
**Comorbidities**	Respiratory	4.81 (4.42, 5.23)	4.21 (3.82, 4.65)	4.18 (3.79, 4.61)	1.89 (1.67, 2.14)
	Circulatory	4.19 (3.86, 4.54)	3.61 (3.27, 3.99)	3.67 (3.32, 4.06)	1.39 (1.21, 1.58)
	Any Cancer	1.69 (1.55, 1.84)	1.49 (1.33, 1.66)	1.49 (1.33, 1.67)	0.55 (0.48, 0.63)
	Type 2 Diabetes	2.71 (2.43, 3.02)	1.90 (1.66, 2.18)	1.98 (1.73, 2.27)	0.53 (0.45, 0.63)
	Kidney	5.10 (4.47, 5.82)	3.90 (3.33, 4.57)	4.05 (3.45, 4.75)	1.09 (0.91, 1.32)
	Liver	4.39 (3.71, 5.19)	3.52 (2.89, 4.28)	3.68 (3.02, 4.48)	1.06 (0.85, 1.33)
	Autoimmune	3.24 (2.89, 3.63)	2.59 (2.27, 2.97)	2.65 (2.31, 3.03)	0.86 (0.74, 1.01)

Abbreviations: OR, odds ratio; n_0, sample size for tested patients; n_0, sample size for comparative group; BMI, body mass index; REF, reference group; SES, social economics status; NDI, 2010 Neighborhood Socioeconomic Disadvantage Index; adjustment 1, age + sex + race; adjustment 2, adjustment 1 + NDI; adjustment 3, adjustment 2 + comorbidity score.

eTable 7. Number of Key COVID-19 Symptoms within 14 days Before the First Test.

		Tested (N=5698)			Tested positive (N=1139)		
		All (n=4830) *	White (n=3234)	Black (n=916)	All (n=989)	White (n=445)	Black (n=381)
Cough	Cough	1568 (32.5)	997 (30.8)	319 (34.8)	360 (36.4)	148 (33.3)	146 (38.3)
	Shortness of breath	1465 (30.3)	913 (28.2)	331 (36.1)	385 (38.9)	150 (33.7)	161 (42.3)
	Other symptoms of respiratory system	2392 (49.5)	1506 (46.6)	509 (55.6)	570 (57.6)	229 (51.5)	233 (61.2)
Fever & chill	Fever of unknown origin	1069 (22.1)	674 (20.8)	233 (25.4)	342 (34.6)	141 (31.7)	137 (36.0)
	Chills (without fever)	30 (0.6)	18 (0.6)	8 (0.9)	6 (0.6)	2 (0.4)	3 (0.8)
Loss of smell and taste	Anosmia	2 (0.0)	1 (0.0)	0	2 (0.2)	1 (0.2)	0
	Other disturbances of smell and taste	2 (0.0)	2 (0.1)	0	1 (0.0)	1 (0.2)	0
	Unspecified disturbances of smell and taste	1 (0.0)	0	1 (0.1)	1 (0.0)	0 (0.0)	1 (0.3)
Pain	Pain in joint	286 (5.9)	199 (6.2)	51 (5.6)	53 (5.4)	26 (5.8)	16 (4.2)
	Symptoms of the muscles	7 (0.1)	4 (0.1)	1 (0.1)	0	0	0
	Nonspecific chest pain	446 (9.2)	257 (7.9)	137 (15.0)	81 (8.2)	25 (5.6)	42 (11.0)
	Tension headache	11 (0.2)	8 (0.2)	3 (0.3)	3 (0.3)	2 (0.4)	1 (0.3)
	Other headache syndromes	541 (11.2)	375 (11.6)	108 (11.8)	94 (9.5)	49 (11.0)	36 (9.4)

*n represents the number of patients with available symptom data.

© 2020 Gu T et al. *JAMA Network Open.*
